# The interplay of age, gender and amyloid on brain and cognition in mid-life and older adults

**DOI:** 10.1038/s41598-024-78308-3

**Published:** 2024-11-08

**Authors:** Léonie Borne, Renate Thienel, Michelle K. Lupton, Christine Guo, Philip Mosley, Anna Behler, Joseph Giorgio, Robert Adam, Amelia Ceslis, Pierrick Bourgeat, Amir Fazlollahi, Paul Maruff, Christopher C. Rowe, Colin L. Masters, Jurgen Fripp, Gail A. Robinson, Michael Breakspear

**Affiliations:** 1 School of Psychological Sciences, College of Engineering, Science and the Environment, University of Newcastle, Callaghan, NSW Australia; 2 School of Medicine and Public Health, College of Health, Medicine and Wellbeing, University of Newcastle, Callaghan, Australia; 3 QIMR Berghofer Medical Research Institute, Brisbane, QLD Australia; 4 ActiGraph LLC, Pensacola, FL USA; 5 CSIRO Health and Biosecurity, Brisbane, QLD Australia; 6 Helen Wills Neuroscience Institute, University of California, Berkeley, CA 94720 USA; 7 UQ Centre for Clinical Research (UQCCR), University of Queensland, Brisbane, QLD Australia; 8 Queensland Brain Institute & School of Psychology, University of Queensland, Brisbane, QLD Australia; 9 Florey Institute, University of Melbourne, Melbourne, VIC Australia; 10 Department of Molecular Imaging & Therapy, Austin Health, Heidelberg, VIC Australia

**Keywords:** Neuroscience, Cognitive ageing, Computational neuroscience, Alzheimer's disease

## Abstract

Deficits in memory are seen as a canonical sign of aging and a prodrome to dementia in older adults. However, our understanding of age-related cognition and brain morphology occurring throughout a broader spectrum of adulthood remains limited. We quantified the relationship between cognitive function and brain morphology (sulcal width, SW) using three cross-sectional observational datasets (PISA, AIBL, ADNI) from mid-life to older adulthood, assessing the influence of age, sex, amyloid (Aβ) and genetic risk for dementia. The data comprised cognitive, genetic and neuroimaging measures of a total of 1570 non-clinical mid-life and older adults (mean age 72, range 49–90 years, 1330 males) and 1365 age- and sex-matched adults with mild cognitive impairment (MCI) or Alzheimer’s disease (AD). Among non-clinical adults, we found robust modes of co-variation between regional SW and multidomain cognitive function that differed between the mid-life and older age range. These cortical and cognitive profiles derived from healthy cohorts predicted out-of-sample AD and MCI. Furthermore, Aβ-deposition and educational attainment levels were associated with cognition but not SW. These findings underscoring the complex interplay between factors influencing cognition and brain structure from mid-life onwards, providing valuable insights for future research into neurodegeneration and the development of future screening algorithms.

## Introduction

Aging is accompanied by substantial cognitive and neuronal changes that reflect the direct influence of neurodegenerative processes and accumulating risk factors, such as chronic cardiovascular and metabolic disease. These age-related changes in brain and cognition are challenging to identify as the cognitive and anatomical variability between individuals is often larger than the age-related differences across a cross-sectional study^1^.Distinguishing healthy aging from the impact of neurodegenerative diseases, such as Alzheimer’s disease (AD), poses a significant challenge^2–4^. The preclinical phase of AD may initiate several decades before formal diagnosis, often commencing in mid-life^5^. Accurately identifying this early stage holds the potential to unveil new diagnostic and therapeutic opportunities^6,7^. However, achieving this requires a nuanced differentiation from the natural aging process spanning a wide range of adulthood.

Healthy aging in older adults is accompanied by both grey and white matter changes^8–10^, with inconclusive sex effects on both, grey^11^ and white matter aging^12,13^. Some regions, such as the prefrontal cortex, appear particularly sensitive to the aging process, while others, such as the medial temporal cortex or the hippocampus, are relatively preserved^14,15^. Lateral prefrontal changes are associated with a decline in executive function^16,17^, whilst neurodegenerative changes in the early stages of Alzheimer’s disease occur predominantly in the hippocampus, precuneus and medial temporal lobe, often manifesting as memory loss^18–22^. Several risk factors for AD have been identified, including age, genetic factors such as the *APOE* ε4 allele^23^ and modifiable risk factors such as fewer years of education, hearing loss, and others^24^. According to the amyloid cascade hypothesis, neurodegeneration in AD is caused by abnormal accumulation of amyloid beta (Aβ) plaques^25,26^, which are composed of amyloid beta (Aβ) peptides^27^. These changes occur well before other biomarker changes, including neurofibrillary tangles. For example, tau mediated neuronal injury^28^, may initiate structural brain changes, then cognitive changes which later lead clinical impairments^29^. While these distinct pathways are extensively documented, the process by which they manifest from the healthy mid-life brain remains unclear.

There is a gap in the literature regarding cognitive and brain changes in mid-life^4^. Large multimodal studies of AD, such as the Alzheimer’s Disease Neuroimaging Initiative (ADNI)^30^and the Australian Imaging, Biomarkers and Lifestyle (AIBL)^31^ focus on the decades for peak onset of AD, i.e. 60–80 years, after the first neurobiological changes of AD, which likely begin several decades earlier^5,7^. The Prospective Imaging Study of Aging: Genes, Brain and Behaviour (PISA)^32^, is a multimodal study including amyloid PET scans that studies midlife aging (mean age 61, range 49–73), with a focus on individuals at high genetic risk of developing AD (*APOE* ε4 positive as well as those with high polygenic risk scores, AD-PRS). An integrative analysis of these large cohort studies would thus enable a cross sectional snapshot of brain and cognition across a wide aperture of mid-life and older adulthood.

Here, we investigate and compare the relationship between cognition and brain morphology in cross-sectional data for two age ranges: one (using PISA) to focus on the mid-life age range and the other (using ADNI and AIBL) focussed on an older age-range corresponding to the typical onset of AD. We use a multivariate method, the canonical Partial Least Square^33^ which enables a global and unbiased analysis of the relationship between brain and behaviour in these three cohorts. For the measure of brain anatomy, we use sulcal width (SW) derived from structural magnetic resonance imaging (sMRI). SW is a promising marker for the sensitive detection of disease which has been shown to be more accurate than cortical thickness (CT) in differentiating mild cognitive impairment (MCI) and AD from healthy people^34^. This advantage is likely due to reduced susceptibility for age-related deterioration of sMRI contrast between grey and white matter^35^. We then examine how brain-cognition relationships are modified by age, sex, education, cortical amyloid and genetic risk factors for AD across these three cohorts. To explore putative relationships between aging and neurodegeneration, we benchmark the analyses of all three healthy cohorts against clinical cohorts of age- and sex-matched persons with MCI or AD.

## Results

We analyzed cognitive, neuroimaging (MRI, PET) and genetic data from 1570 healthy adults (healthy cohorts, HC) and 1365 adults with MCI or AD (clinical cohorts, CC), drawn from 3 multimodal databases; PISA, ADNI and AIBL. While the age ranges of these cohorts mutually overlapped, the PISA cohort spanned a younger mid-life cohort than AIBL and ADNI (Fig. S1). To integrate brain and cognitive data, we used partial least squares (PLS), a multivariate method that identifies modes of covariation between two multivariate data set, here regional SW and multidomain neurocognitive scores (see Methods). Nonparametric testing was performed to identify robust modes of covariation (with a Bonferroni corrected statistical threshold set at *p* < 0.004).

### Brain-behaviour modes in mid-life *PISA* participants

Application of PLS to the 190 healthy PISA HC participants, using SW as the anatomical measure, yielded a single robust mode (1st mode, *p* < 0.001, *z*-covariance = 5.49; 2nd mode, *p* > 0.99). Using cortical thickness (CT) as the brain measure also yielded a single mode, but with less covariance explained (1st mode, *p* = 0.0060, *z* = 3.07; 2nd mode, *p* > 0.99). We hereafter focus on the SW-derived mode as it explains greater than 50% more brain-behaviour covariance in all PLS analyses (see Supp. Fig. S2 for the CT-derived PLS analyses).

**Fig. 1:**
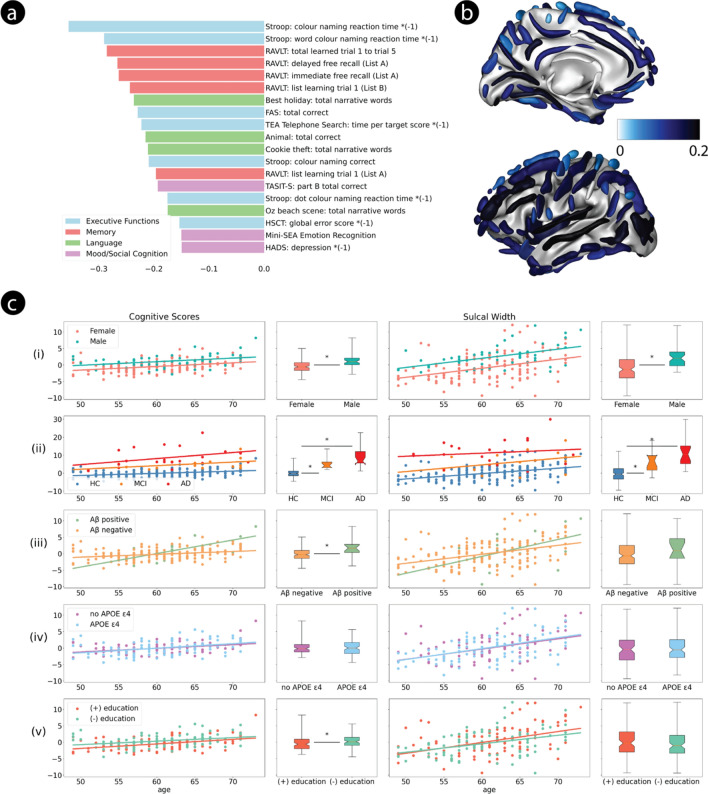
Robust PLS mode trained on PISA healthy cohort. (**a**) Mean loading of all reliable cognitive scores, colour-coded by domain. Larger (negative) projections denote poorer performance. (**b**) Mean loading of all reliable cortical sulci. Higher projections (darker blue) denote wider sulci. (**c**) Effect of age on PISA brain-behaviour mode. Cognitive (left) and sulcal width (right) projections of the single robust mode. More positive projections denote poorer cognitive performance and wider sulci, respectively. (i) Healthy male (cyan) and female (orange) participants, (ii) Healthy amyloid positive (green) or negative (orange) participants, (iii) Healthy cohort (HC, blue) and participants with MCI (orange) or AD (red), (iv) Healthy participants with (blue) or without (purple) the allele 4 of the *APOE* gene, (v) Healthy participants with years of education above (red) or below (green) the median. Lines and scatter plots show the evolution of the projections as a function of age. The box plots show the distribution of the cognitive and brain projections by group. The box extends from the lower to upper quartile values of the data, with a notch at the median. The whiskers extend from the box from the minimum to the maximum values. The influence of groups on the projection is evaluated with an ANCOVA controlling for age (i) and age and sex (ii–v). Significant at *p* < 0.004 (Bonferroni corrected).

This robust PLS mode loaded across three main cognitive domains, with the strongest contribution of tests of executive function (Fig. 1a). The brain projection of the mode (SW, Fig. 1b) loaded most strongly onto the occipital lobe, the intraparietal sulcus, the posterior inferior temporal sulcus, the posterior lateral fissure, and the sub-parietal sulcus (Supp. Fig. S2). Both the cognitive and brain projections were strongly correlated with the age of the participants (SW, *p* = 6.1e–9; cognition, *p* = 8.7e–7). The projections also differed as a function of sex (Fig. 1c left), which persists when controlling for age (SW, *p* = 1.2e–6; cognition, *p* = 1.6e–6; Fig. 1c right).

We applied this PLS model—trained on healthy participants—to the 35 age- and sex- matched MCI and AD PISA participants (Fig. 1c). The brain and cognitive projections loaded more strongly with disease stage—i.e. are significantly higher for AD than for MCI and in turn for MCI compared to HC. Furthermore, there was a significant interaction between age-and clinical status where the HC group had greater differences in cognition between younger and older participants (*p* = 0.022). Among healthy participants, the cognitive score was significantly influenced by the presence of amyloid (*p* = 0.0019) but not by the *APOE* ε4 allele (*p* = 0.69). Specifically, older amyloid positive participants had poorer cognitive performance, compared to amyloid negative participants (*p* = 6.6e–04). The SW score was not associated with either of these factors (amyloid, *p* = 0.85; *APOE*, *p* = 0.55; Fig. 1c).

### Brain-behaviour modes in older adult AIBL participants

**Fig. 2:**
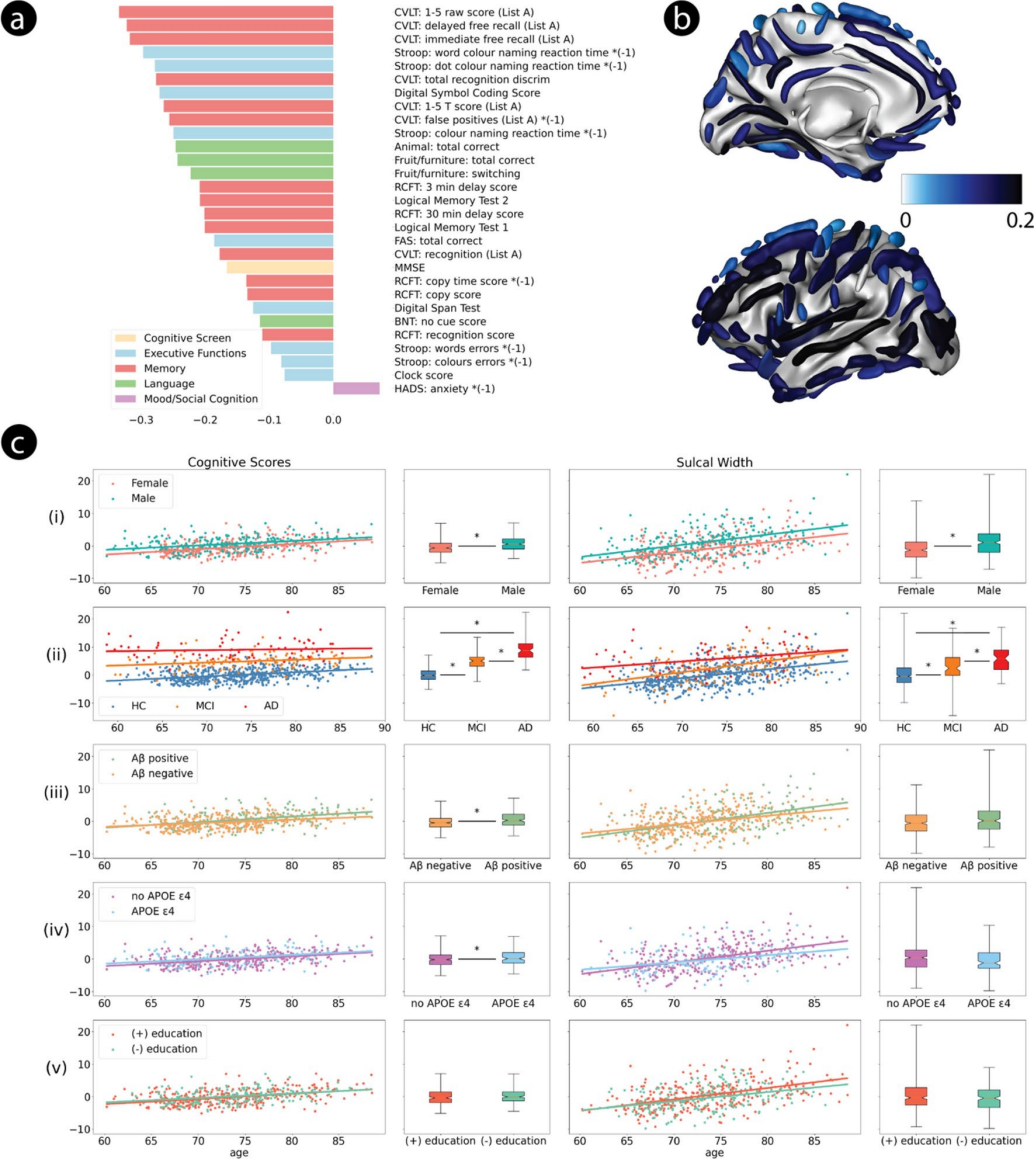
Robust PLS mode trained on AIBL healthy cohort. (**a**) Mean loading of all reliable cognitive scores. (**b**) Mean loading of all reliable cortical sulci. (**c**) Effect of age on AIBL brain-behaviour mode. Panels and legend as per Fig. 1.

Application of PLS to the 573 healthy older adult AIBL participants also yielded a single robust mode (*p* < 0.001, *z* = 6.60). As with the PISA data, this single SW-derived mode loaded across all three main cognitive domains but, notably, with strongest affinity for the memory domain, not executive function (Fig. 2a). The brain projection loaded most strongly over the superior temporal sulcus, the posterior lateral fissure, the inferior frontal sulcus, the occipital lobe and the intermediate frontal sulcus (Fig. 2b; Supp. Fig. S2). Interestingly, the first mode was not robust when using CT as the brain measure (*p* = 0.11).

As with the PISA data, cognition and SW scores correlated significantly with age (SW, *p* = 9.7e–29; cognition, *p* = 1.0e–19) and also with sex, when controlling for age (SW, *p* = 4.2e–13; cognition, *p* = 9.0e–11, Fig. 2c). The PLS model trained on healthy participants, controlling for age and gender, again loaded more strongly onto the 191 AD than MCI participants, and more strongly for MCI than for HC participants (Fig. 2c). Furthermore, there was an interaction between age and clinical status with HC seeing greater differences in cognition for younger and older participants than AD participants (*p* = 0.0053). Of note, the cognitive projection was significantly higher in the presence of both amyloid (*p* = 0.0012) and the *APOE* ε4 allele (*p* = 0.0039, Fig. 2c). The SW projection is not influenced by either of these factors.

### Brain-behaviour modes in older adult ADNI participants

**Fig. 3:**
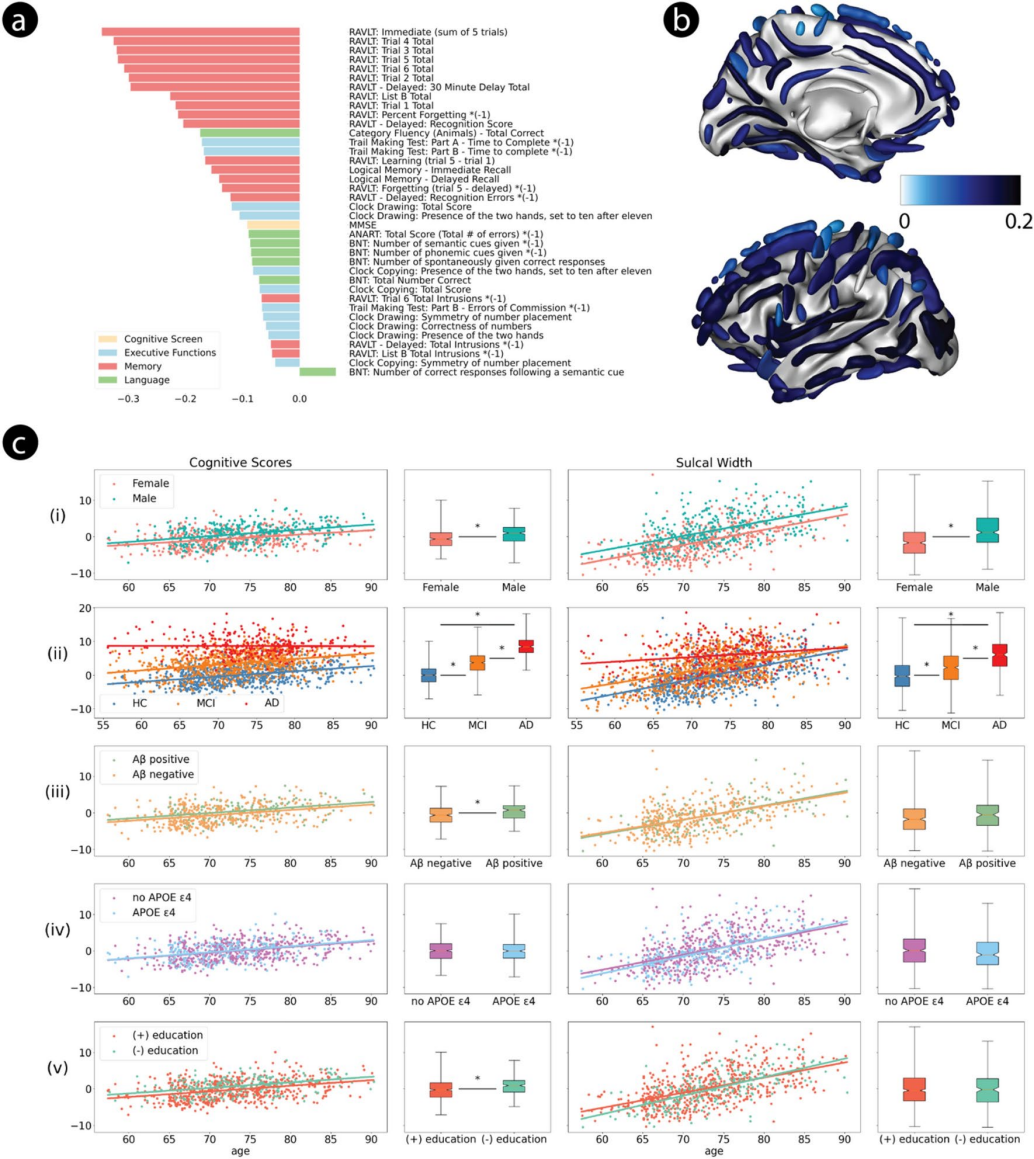
Robust PLS mode trained on ADNI healthy cohort. (**a**) Mean loading of all reliable cognitive scores. (**b**) Mean loading of all reliable cortical sulci. (**c**) Effect of age on ADNI brain-behaviour mode. Panels and legend as per Fig. 1.

Application of PLS to the 807 healthy older adult participants from the ADNI cohort also yielded a single robust mode (*p* < 0.001, *z* = 12.67). The cognitive projection loadings showed effects comparable to the older AIBL cohort, covering all domains, with strongest loadings on tests of the memory domain (Fig. 3a). The brain anatomical projections were strongest over the posterior inferior temporal sulcus, the occipital lobe, the collateral fissure, the insula and the anterior inferior temporal sulcus (Fig. 3b; Supp. Fig. S2). Similar to the PISA data, using CT as the brain measure yielded a single mode that explained less *z*-covariance (*p* < 0.001, *z* = 4.88, Supp. Fig. S4).

As with the PISA and AIBL data, both the cognitive and anatomical projections were correlated with age of the ADNI participants (SW *p* = 4.2e–68; cognition, *p* = 3.0e–26) and differed for sex, when controlling for age (SW, *p* = 7.9e–19; cognition, *p* = 3.7e–10; Fig. 3c). When comparing with the clinical cohort (n = 1139), whilst controlling for age and sex, both projections were significantly higher for AD than for MCI and for MCI compared to HC participants (Fig. 3c). Furthermore, there were interactions between group and age between AD and HC (SW, *p* = 1.8e–11; cognition, *p* = 1.8e–8), between MCI and AD (SW, *p* = 1.6e–6; cognition, *p* = 1.6e–7) and for the brain projections between MCI and HC participants (SW, *p* = 0.034; cognition, *p* = 0.54). The cognitive projection was significantly influenced by the presence of amyloid (*p* = 9.6e–4) but not by the *APOE* ε4 allele (*p* = 0.31). The SW projection was not influenced by either of these factors (amyloid, *p* = 0.64; *APOE*, *p* = 0.56) (Fig. 3c).

### Brain-behaviour modes across healthy and clinical participants

We next included the PISA clinical participants (with MCI or AD) alongside the healthy PISA participants, effectively increasing the cognitive variance into the clinically impaired range. Using SW as the anatomical measure and regressing out age and sex, yielded a single robust PLS mode (1st mode, *p* < 0.001, z = 16.3; 2nd mode, *p* > 0.99). Likewise using CT as the anatomical measure yielded a single mode but with less covariance explained (1st mode, *p* < 0.001, z = 10.6; 2nd mode, *p* > 0.99; Supp. Fig. S5).

**Fig. 4:**
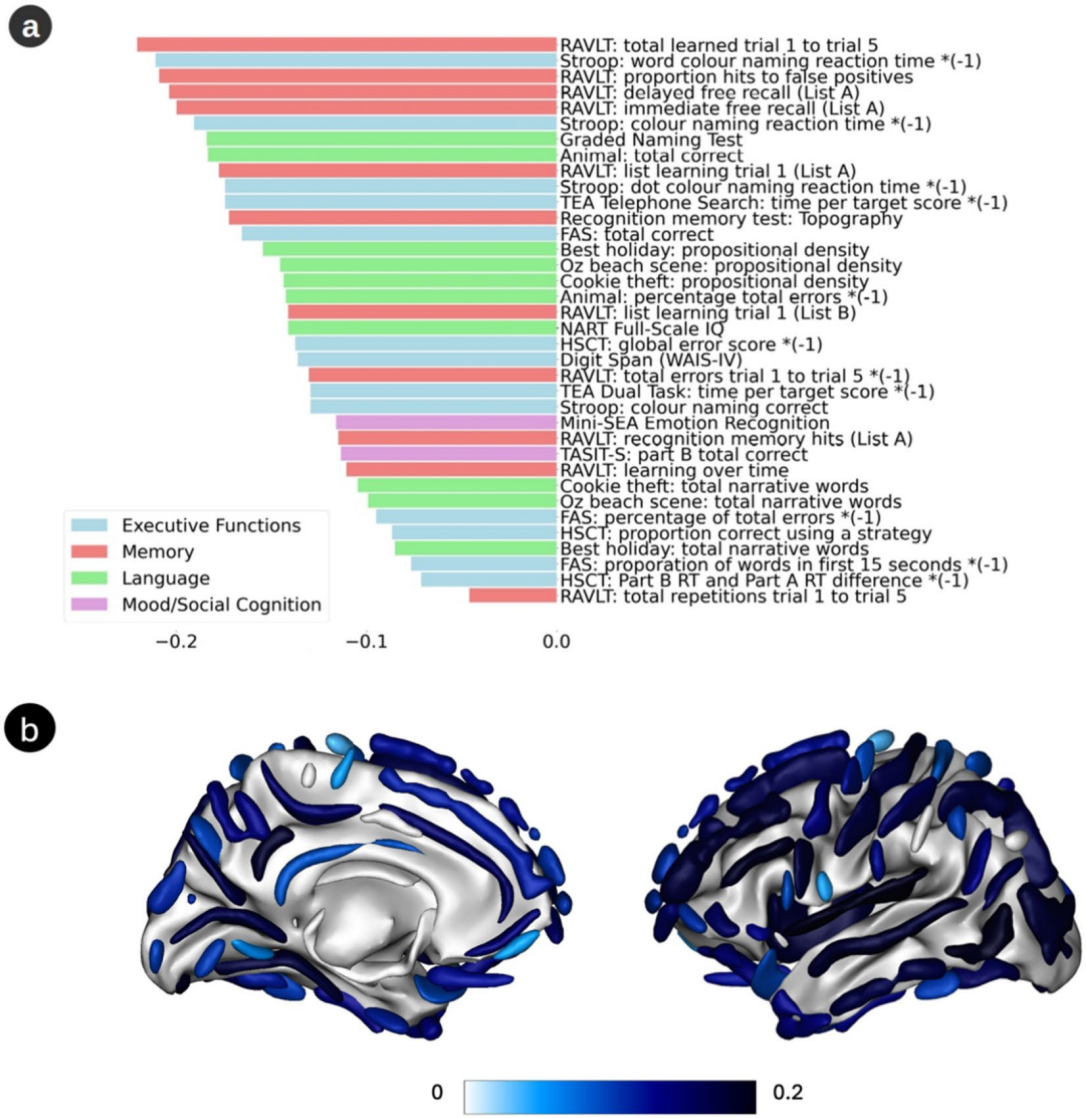
Robust PLS mode trained on the PISA cohort with both health (HC) and clinical (CC) participants. (**a**) Mean loading of all reliable cognitive scores. (**b**) Mean loading of all reliable cortical sulci.

Compared to the PLS model trained only on the PISA HC data, the robust mode including clinical participants loaded with greater affinity onto the memory tests (Fig. 4a). The brain projection loaded most strongly with the SW of the posterior inferior temporal sulcus, the sub-parietal sulcus, the posterior lateral fissure, the superior temporal sulcus and the occipital lobe (Fig. 4b; Supp. Fig. S2). The cognitive projection was higher for healthy amyloid positive participants (*p* = 0.0027) but was not significantly influenced by the presence of *APOE* ε4 allele (*p* = 0.28). The brain projection was not significantly influenced by the presence of amyloid (*p* = 0.99) or the presence of *APOE* ε4 allele (*p* = 0.53).

**Fig. 5:**
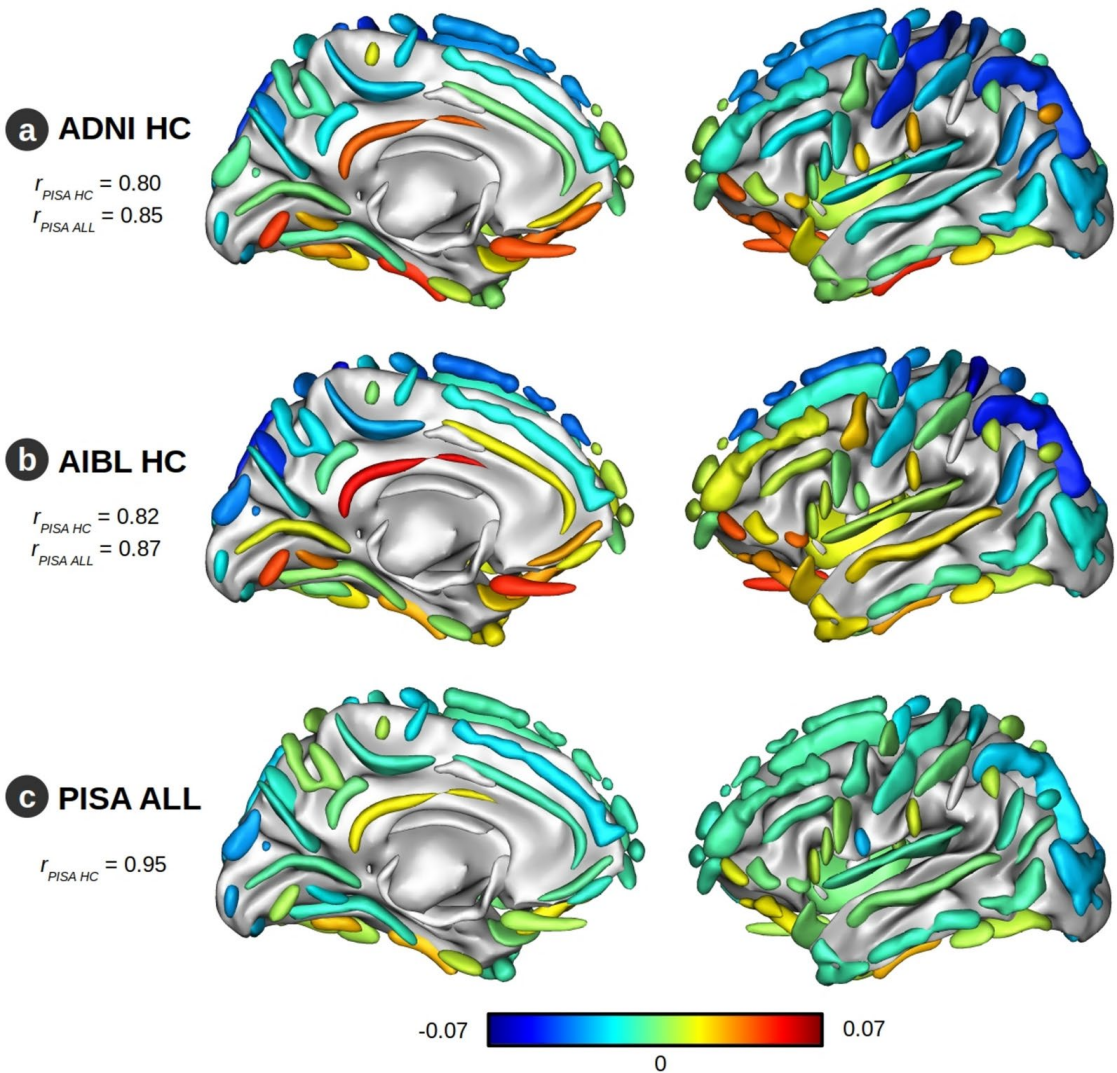
Brain loadings correlations and differences. (**a**) Differences in the SW loadings between the healthy ADNI cohort (ADNI HC) and the healthy PISA cohort (PISA HC). Positive values imply that the SW loadings of PISA HC are lower than those of ADNI HC. (**b**) Corresponding differences between the AIBL HC and PISA HC. (**c**) Difference between PLS trained on all PISA participants, after regression out of age and sex (PISA ALL) and healthy participants (PISA HC). The Pearson correlation coefficients (*r*) are shown between the brain loadings of the specified cohort (ADNI HC; AIBL HC; PISA ALL) and those of PISA HC (*r*_*PISA HC*_) and PISA ALL (*r*_*PISA ALL*_).

Although the exact composition of the cognitive tests across the three studies differed, it is notable that the inclusion of clinical participants in the PISA PLS model shifted the cognitive loading with now strongest loadings onto tests of memory function, as opposed to executive function. Comparing the anatomical loadings across the cohorts (Fig. 5) shows that they bear stronger similarity when comparing the PISA model, including clinical participants, with the healthy AIBL/ADNI models, than when comparing the PISA model, including only (younger) HC participants, with the same AIBL/ADNI models.

### Influence of hippocampal volume on cognition

Considerable prior work has focussed on the role of changes in hippocampal volume in aging and neurodegeneration^36–38^. We hence sought to benchmark the relative influence of hippocampal volume (HV) against cortical sulcal width on cognition in the PISA cohort. Complimenting the list of cortical SWs by including left and right HV had negligible impact on the ensuing robust mode. Specifically, this mode was similar to the mode derived without inclusion of the HVs, with no significant impact on the cognitive projection (95% confidence interval: left HV {− 0.03, 0.04}; right HV {− 0.02, 0.06}).

**Fig. 6:**
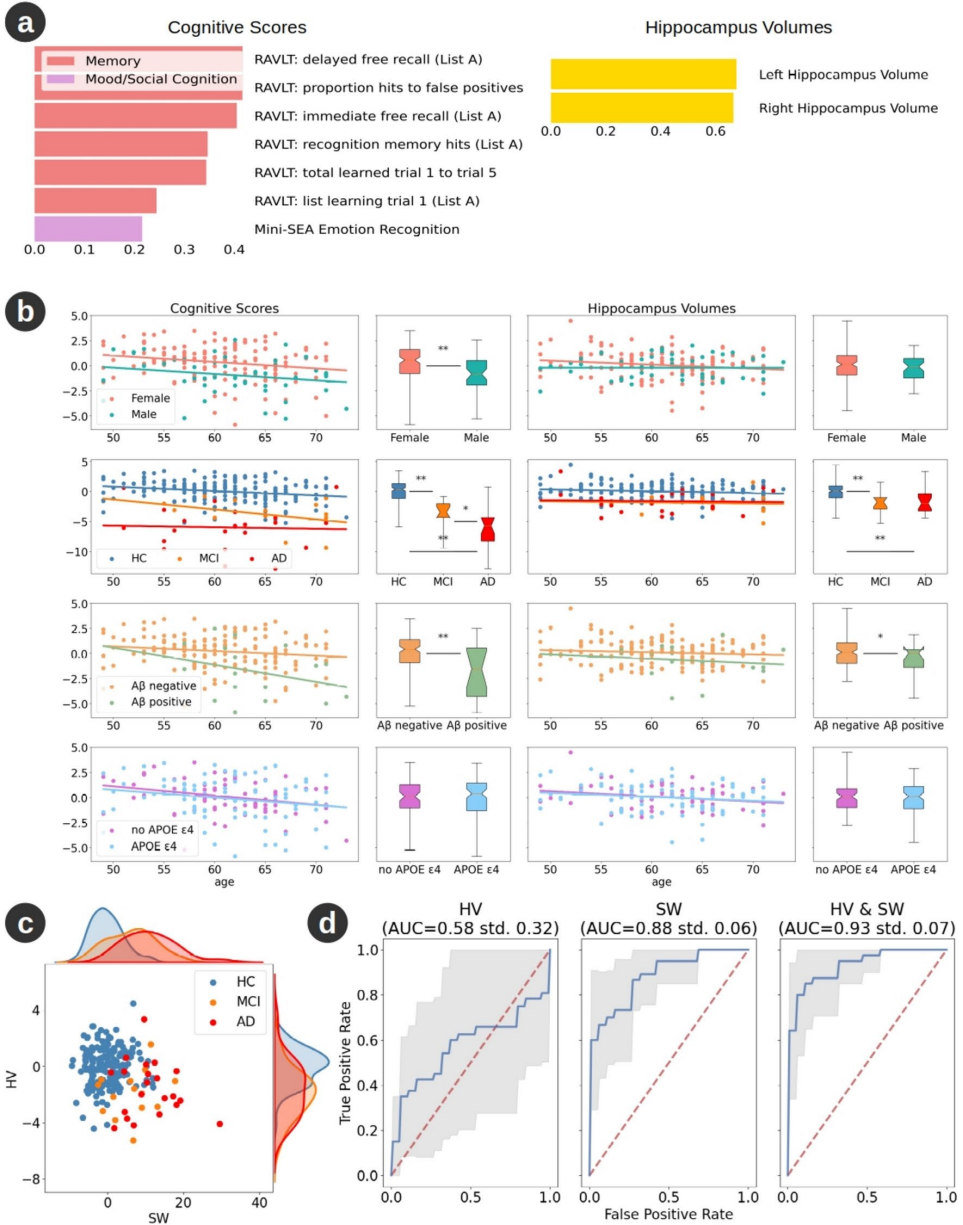
PLS mode trained on the healthy PISA cohort using hippocampal volume (HV) as the anatomical measure. (**a**) Mean loading of all reliable cognitive scores and of left and right HV. (**b**) Effect of age on HV-derived PISA brain-behaviour mode. Panels and legend as per Fig. 1. Note that greater expression of this mode (more positive values) coincides with better performance of the corresponding cognitive loading (i.e. better performance on memory tests) and larger hippocampal volumes. (**c**) Comparison of hippocampus volume (HV) and sulcal width (SW) projections between PISA healthy cohort (blue) and participants with MCI (orange) or AD (red). Larger sulcal width covaries with smaller hippocampal volumes across participants. (**d**) Area under the ROC curve (AUC) after training a linear support vector machine to classify healthy and clinical participants, using a stratified tenfold cross validation, based on the HV and/or the SW projections. The SW mode more accurately differentiates between HC and CC participants than the HV-derived mode. The use of both SW and HV scores provides a slightly better differentiation between the two cohorts. Note that these classification models are based on anatomical measures only. Supplementary Fig. 6 shows the results when age, sex, education and APOE status are included.

Application of the PLS only on the HVs yielded a single robust mode that is almost exclusively correlated with the memory tests (Fig. 6a) but explained relatively little cognitive covariance (*p* = 0.050, *z* = 1.73). In contrast to SW, the HV projections were not correlated with age (HV, *p* = 0.087; cognition, *p* = 0.0023), did not differ neither by sex (HV, *p* = 0.26; cognition, *p* = 8.1e–5) nor differentiate MCI from AD participants (HV, *p* = 0.76; cognition, *p* = 0.038). However, the mode was significantly influenced by amyloid status (HV, *p* = 0.037; cognition, *p* = 5.5e–5; Fig. 6b) and did differentiate HC’s from MCI. Thus, amyloid accumulation in healthy subjects seemed to be disproportionally related to HV loss rather than SW increase. However, we found no significant difference across age bands on HV projections between Aβ + and Aβ- participants (HV, *p* = 0.75; cognition, *p* = 0.18).

We next formally compared the differentiation of HC from AD and MCI using whole brain SW versus HV (Fig. 6c, d; Supp. Fig. S6). Both SW and HV projections differentiated HC from AD and MCI. However, the SW projection was more accurate than HV. The combination of SW and HV was more accurate than either considered alone, suggesting a degree of independence in these metrics.

### Healthy aging and neurodegeneration

Although the preceding PLS modes from all three cohorts do not explicitly model age effects in our cross-sectional samples, the influence of age in all cohorts is consistently strong, reflecting the strong influence of aging on cognitive and anatomical variance across the age-range of these cohorts. These modes comprise multi-domain poorer cognitive performance and widespread wider sulci that also reflect individual variability. This raises the question of whether the specific composition of these modes are implicitly optimized to covary with age, or whether any linear weighting of poor cognition and wider sulci would perform comparably well. To test this, we randomly permuted the (SW and cognitive) mode features, breaking the specific association of each feature with its loading, hence producing surrogate PLS modes comprised of linear combinations of randomly chosen features. Performing this permutation 1000 times yields a reference distribution for non-specific anatomical and cognitive variability across our cohorts.

**Fig. 7:**
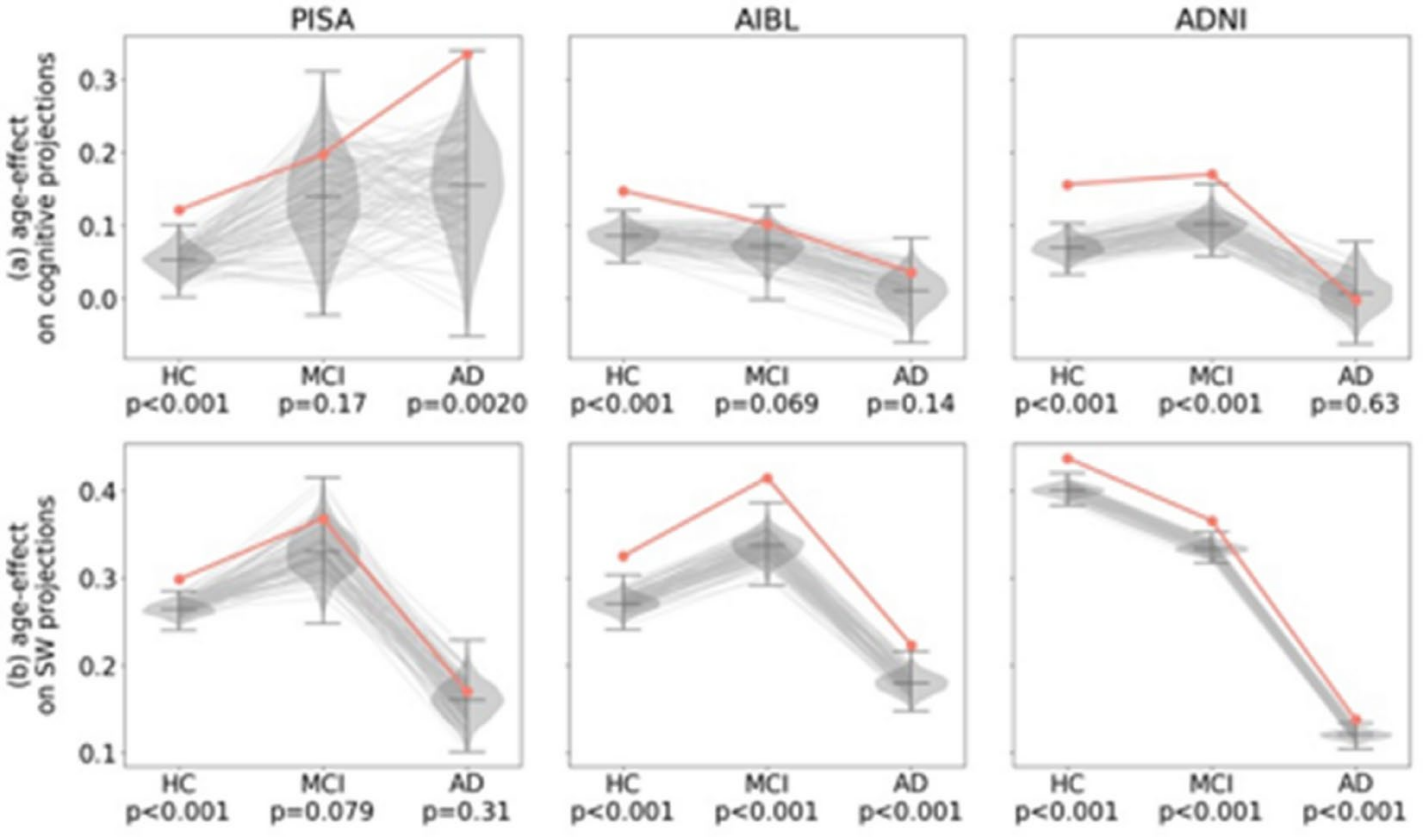
Specific versus non-specific age and diagnostic effects. Each column represents a different cohort. Results for the slope of the linear regression of the cognitive (**a**) and brain (**b**) projections against age (red) compared to randomly chosen features (grey). See Supplementary Fig. S7 for results for the cognitive  (**a**) and brain (**b**) projections for HC, MCI and AD participants, also benchmarked against randomly chosen features (grey). The violin plots represent the distribution of scores for 1000 permutation tests (bars represent the minimum, median and maximum value). The red line represents the original scores using PLS. The grey lines represent the scores for 100 representative permutations. The *p*-value is shown under the cohort label. Note that the *p*-value is not shown for the mean value of the healthy cohorts because the mean is always zero.

Performing this test shows that the original feature loadings (Fig. 7a, b; red lines) almost always out performs these surrogates (Fig. 7a, b; grey). These tests show that the PLS trained on HCs in each cohort returns the optimal combination of cognitive and SW weights that covary with age in the HC cohort, when benchmarked against randomly chosen features. This suggests that age-related differences across the cohorts are stronger than inter-individual effects, and (given the loadings differ) that these age-related differences vary between midlife and older adulthood. The age-related differences—trained on the HC participants within each cohort—also generally predicted age-related differences in the MCI and AD participants, noting that in some of the cohorts, the age-related differences in the clinical groups were reduced (such as AD in ADNI).

We then tested whether the anatomical and cognitive features identified by performing PLS on the HC were optimized to identify independent cohorts of participants with MCI and AD, using the same permutation method (Supp. Fig. S7a, b). For all 3 cohorts, the exact combination of sulci always performed superior to randomly chosen sulci (Supp. Fig. S7b). For AIBL and ADNI, the weights of cognitive scores were always superior to randomly selected cognitive tests. For the PISA cohort, the PLS mode trained on the HC ranks cognitive tests that usually, but not always, outperformed randomly chosen tests (Supp. Fig. 7a).

Finally, we correlated the brain projections of all three cohorts using age matched (median age of 65 years) sub-samples of each cohort. Correlations between the SW loadings of all three healthy samples, and the combined PISA sample (HC, MCI and AD) ranged between r = 0.76 (AIBL and PISA), and 0.87 (AIBL and ADNI) as well as 0.91 for PISA ALL and PISA HC (Supplementary Fig. S8).

## Discussion

In sum, this study demonstrates a single mode of covariation between cortical anatomy and cognitive function at midlife, and a single mode in older adulthood. While the cortical loadings are similar across the cohorts, there is a subtle re-ordering of the cognitive loadings, with tests of executive function (e.g. the Stroop test) stronger than memory in the mid-life cohort, switching relative rank in the two older cohorts, in line with our previous work^39^. Age-related effects exerted a strong influence on the expression of these modes, which also differed by gender, amyloid status and the presence of MCI or AD. As a result, the brain-behavior relationships observed in healthy data reflected the patterns associated with MCI and AD diagnoses in matched clinical participants.

Education attainment was significantly associated with the cognitive but not SW projections, indicating an association with cognitive function but not (yet) with the underlying brain structures. Similarly, the presence of amyloid significantly impacted the cognitive projection, but not the SW projections, in all healthy cohorts. Given the substantial sample size (and hence power) of our study, the impact of amyloid on cognition but not SW suggests an impact on incipient neuronal integrity of cognitive relevance, but which does not (yet) impact large-scale cortical anatomy, even in the older cohorts. In contrast, for the hippocampal-derived PLS, amyloid influences both the anatomical (HV) projection and the associated (memory dominated) cognitive projection. This selective impact suggests that amyloid deposition leads to neuronal changes that deviate from those of healthy aging (which influence many cortical regions and executive functions) leading to anatomically specific changes in the hippocampus and related cortical regions, such as the precuneus, with a disproportionate impact on memory^40^.

The presence of the ε4 allele of the *APOE* gene does not impact on the anatomical projections. It impacts cognitive projections for the older (AIBL) but not the mid-life (PISA) population. This may imply that its effect is less important, or even negligible, in mid-life adulthood. However, the PISA database has weaker power than AIBL, which may explain the lack of significant differences, particularly as the effect size in the AIBL data is small. Indeed, power analyses (alpha = 0.05) for the results of the PLS trained on PISA HC show that the power is less than 20% when studying the influence of the *APOE* ε4 allele (SW, power = 18%; cognition, power = 19%).

Age-related differences depend on the age range studied. For the older populations studied (AIBL and ADNI), the influence of age is significantly more pronounced for healthy participants (independently of amyloid level) than for those with MCI or AD. This may be because participants with dementia present advanced brain aging^41,42^, with a ceiling effect that mitigates the additional effect of chronological age. Conversely, for the mid-life population studied (PISA), age-related differences on cognition are weaker for healthy participants than for those with MCI or AD, and similarly for amyloid-negative compared to amyloid-positive healthy participants. Therefore, at mid-life, the early stages of dementia and amyloid accumulation accelerate cognitive aging, whereas for older participants, dementia has already degraded cognition, overwhelming age effects in a cross-sectional study.

The composition and ranking of the cognitive domains varied across cohorts. Executive functions expressed the strongest brain-cognition covariance across the non-clinical mid-life (PISA) cohort, whereas performance on memory tests exerted the greatest influence when also incorporating individuals with early AD (PISA ALL) and in the older (ADNI, AIBL) cohorts. This suggests that memory tests explain individual and age-related differences in brain structure the strongest in older and clinically impaired cohorts potentially due to mild to moderate disease related neurodegeneration. In contrast, anatomical differences in mid-life brain structure are associated with executive function. However, caution is warranted given the lack of harmonization of cognitive test batteries in these three cohorts. Furthermore, the cohorts had varying representation for each cognitive domain as PISA had more executive functioning tests, whereas AIBL and ADNI had more memory tests.

Similar to cognition, the anatomical projections showed subtle variations across cohorts. SW loaded heavily on regions classically susceptible to aging (prefrontal cortex, insula, superior parietal gyri, central sulci, cingulate sulci, calcarine cortex)^14,15,43^ or by early progression of AD (temporal lobe, posterior cingulate, retrosplenial cortex)^44,45^. Comparing AIBL/ADNI HC to PISA HC, suggests that fronto-temporal regions are more strongly involved with cognition in the older population, while occipito-parietal regions are more involved in the mid-life population. Similarly, occipito-parietal regions appear more relevant for PISA HC than when also including the CC. The distinction between fronto-temporal and occipito-parietal regions in explaining cortical aging in relation to cognitive decline has recently been highlighted in Cox et al.^46^, showing that fronto-temporal regions explain more of cortical aging in the eighth decade of life than occipito-parietal regions. Our study also supports this finding and suggests that the occipito-parietal regions explain relatively more of the healthy cortical aging in the seventh decade of life.

Among the different brain measurements studied, sulcal width covaried more strongly with cognitive scores than cortical thickness or hippocampal volumes. Moreover, local anatomical contributions were specified less precisely when using CT than SW. As previously suggested^34^ , two advantages of SW may explain this: 1. Unlike CT, SW measurement does not depend on the grey-white matter boundary that blurs with age^47^; 2. SW incorporates CT thinning as well as white matter reduction around the sulci. Compared to cortical SW, hippocampal volume at mid-life did not show an age effect and differentiated between the healthy cohort and the clinical cohort less accurately than the SW projection. Although surprising, note that the raw and ICV-corrected HV measures were also not significantly correlated with age and did not differentiate MCI subjects from AD subjects. The lack of a significant difference between MCI and AD might be due to the limited size of the clinical cohort. The non-correlation with age is intriguing and suggests that, unlike SW, HV is not impacted by direct associates of aging (neural death) at mid-life but is vulnerable to pathological neurodegenerative processes, such as dementia and amyloid accumulation.

There are several caveats to our study. First, the healthy mid-life population studied (PISA HC) is enriched for people at high genetic risk for AD. Although we did not find a direct influence of the risk-selection criteria (*APOE* status and AD-PRS) on the projections obtained, this selection policy could have influenced the results obtained. Second, each database used different cognitive tests, with a less focussed assessment of executive functions for ADNI. This could explain the subtle difference in cognitive and SW loadings results between PISA and ADNI. Third, averaging across hemispheres inherently risks loss of information. Harmonization across platforms introduces additional challenge to differences in quality control and data ascertainment. For example, our analysis could not focus on subdomains across different samples due to subtle yet significant differences in the neuropsychological testing procedures. Consequently, we opted for the approach of using a general latent cognitive variable to ensure consistency and reliability across the data. Future work within cohorts could seek closer links between specific cognitive domains and regional changes in sulcal width. Fourth, we used a mean imputation approach due to its relative simplicity and ease of use over alternative approaches. We believe that this had negligible effects in the covariance estimation at the core of the PLS estimation. Simulation studies evaluating the effect of proportion of missing variables in a PLS framework show that PLS produces unbiased estimates as long as the proportion of missing data does not exceed 8.89% (DOI: 10.5445/KSP1000098011/04). We had missing observations of 322 out of possible 9450 observations in the PISA sample (3.4%), 745 out of 19,125 in the AIBL sample (3.9%), and 896 out of 50,596 in the ADNI sample (1.7%). As such, the low proportion of mean imputed variables will unlikely bias the estimation of the PLS modes presented in this study. Finally, the cross-sectional nature of the analysis provides only indirect insights into aging, thereby indirectly reflecting potential longitudinal trajectories. Further work is required to see if these age-related snapshots across participants reflect age-related changes within participants scanned longitudinally.

## Methods

Data acquisition and analysis followed approval from the Human Research Ethics committees of The University of Newcastle (H-2020-0158) and was in accordance with the National Statement on Ethical Conduct in Human Research. Written informed consent was obtained from all participants following local institutional ethics approval.

### Participants

Cognitive, neuroimaging (MRI, PET) and genetic data from 1570 healthy adults (healthy cohorts, HC) and 1365 adults with MCI or AD (clinical cohorts, CC) were drawn from 3 multimodal databases; PISA, ADNI and AIBL (Supplementary Fig. 1). All participants had a structural (T1-weighted) MRI scan and at least 50% of the cognitive scores available. The databases were sampled such that participants in each database were matched for age and sex (Supp. Table 1).

#### Prospective imaging study of aging (PISA)

The PISA cohort comprised a mid-life population enriched for high genetic risk of AD, derived from the Prospective Imaging Study of Aging (PISA): Genes, Brain and Behaviour^32^. In addition to this genetically enriched sample, patients meeting formal criteria for MCI/AD across the same age range were recruited from local memory outpatient clinics^32^. AD and MCI diagnosis was ascertained at a clinical consensus meeting based on detailed neuropsychology and multi-dimensional MRI based on current criteria (as previously described^32^). We subsampled 190 healthy mid-age Australians (HC; mean age 61, range 49–73; 49 males) selected to be age- and sex-matched to 35 clinical participants with MCI or early onset AD (CC, mean age 63, range 51–72; 15 males). All data were acquired at a single site (Brisbane, QLD).

#### Australian imaging, biomarker & lifestyle flagship study of aging (AIBL)

The AIBL cohort comprised older adults from the Australian Imaging, Biomarker & Lifestyle (AIBL) Flagship Study of Aging (www.aibl.csiro.au). Data was collected by the AIBL study group. AIBL study methodology has been reported previously^31^. We selected 573 healthy participants ascertained at AIBL baseline (mean age 73, range 60–89; 255 males) and 191 age- and sex-matched participants meeting criteria for MCI or AD (mean age 74, range 59–85; 101 males). A consensus diagnosis was assigned at clinical review based on current diagnostic criteria (as previously described^31^). MRI data were acquired at two centres (Perth, WA; Melbourne, Vic) on 7 different Siemens scanners. Only scanners with greater than three participants were included.

#### Alzheimer’s disease neuroimaging initiative (ADNI)

The ADNI cohort focuses on an older American healthy population, derived from the Alzheimer’s Disease Neuroimaging Initiative (ADNI) database (adni.loni.usc.edu). We selected 807 healthy participants (mean age 73, range 57–90; 356 males) and 1139 age- and sex-matched participants meeting criteria for MCI or AD (mean age 73, range 56–89; 554 males). A detailed description of the diagnostic criteria can be found on the ADNI website (https://adni.loni.usc.edu/data-samples/adni-data/study-cohort-information/). Data was acquired at 67 different sites. Only sites with greater than three participants were included. The ADNI data have been curated and converted to Brain Imaging Data Structure (BIDS) format^48^ using Clinica^49,50^.

### Structural and molecular neuroimaging

***Brain:*** T1-weighted structural Magnetic Resonance Imaging (sMRI) data were used to study brain anatomy. Structural data were acquired using a 3D-MPRAGE sequence (see scanner information in Supp. Method).

Positron emission tomography (PET) data were used to quantify amyloid status (positive or negative) of all participants. For the PISA participants, PET data were acquired on a Biograph mMR hybrid scanner (Siemens Healthineers, Erlangen, Germany) with ^18^F-florbetaben, a diagnostic radiotracer which possesses a highly selective binding for β-amyloid in neural tissue^51,52^. Amyloid data for the ADNI and AIBL cohorts were assessed with one of the five following tracers: ^11^C-Pittsburgh Compound B, ^18^F-florbetaben, ^18^F-florbetapir, ^18^F-flutemetamol or ^18^F-NAV4694. The CapAIBL software (Bourgeat et al., 2018) was used to quantify each image into centiloids (CL) allowing the classification of the participants as amyloid positive (> 20 CL) or negative (< 20 CL). Non-negative matrix factorisation was used to improve centiloid robustness across tracers and scanners^53^. Amyloid data were not available on 17 of the HC PISA participants, 4 AIBL HC participants and 444 of the ADNI HC participants.

***Cognition:*** Cognitive and mood assessments were conducted by trained neuropsychologists at all sites. Participants completed a battery of standardized tests selected to assess multidomain cognitive functions (memory, language, visuospatial, attention, processing speed, social cognition and executive function;^32,54,55^). These tests were grouped into four categories (memory, language, executive functions and other, Supp. Table 2 & missing data in Supp. Figure 9–11).

***APOE ε4: APOE*** genotype was determined from blood-extracted DNA^31,32,54^. The *APOE* genotype was not available in 13% of the HC AIBL participants and 3% of the HC ADNI participants. Among the HC participants whose *APOE* genotype was available, the proportion of those with at least one ε4 allele of the *APOE* gene was 53% of the PISA participants, 29% of the AIBL participants and 32% of the ADNI cohort.

### Data processing and modelling

#### Sulcal width (SW), cortical thickness (CT)

The Morphologist pipeline of the BrainVISA toolbox^56^ was used to extract local measures of brain anatomy (see Supp. Method for processing and QC details). The pipeline was applied in a docker image as described in https://github.com/LeonieBorne/morpho-deepsulci-docker. This pipeline identifies 127 cortical sulci, 63 in the right hemisphere and 64 in the left hemisphere. We extracted both cortical thickness (CT) around each sulcus and the sulcal width (SW), which have both shown potential for the early detection of AD^34,57^. As in Dauphinot et al., (2020), right and left hemisphere measurements are averaged when the same two sulci exist on each hemisphere, resulting in 64 unique measurements (see Supp. Figure 12 for abbreviations and full labels). We then used ComBat, a technique adopted from the genomics literature^58^ and recently applied to cortical thickness data^59^, to combine and harmonize the sulcal measurements across acquisition sites while preserving age, sex and diagnosis covariates. Three sulci were missing in more than 50% of participants (S.GSM., F.C.L.r.sc.ant., S.intraCing) and were not used in further analyses.

For the PISA database, hippocampal volume was estimated separately for a comparative analysis. The volume of the right and left hippocampi were estimated using the CurAIBL platform^60^ and corrected by dividing by the intracranial volume (ICV) and multiplying by the average ICV of all participants. Although other approaches exist, this method returns comparable results to Freesurfer^60^. Using this AIBL-standard method allows stable assessment across prior reported studies on hippocampal volume from the AIBL study. (see Supp. Method for processing details).

#### Cognitive and mood scores

Each cognitive score was signed so that a more positive value indicated better performance (e.g. task accuracy) and more negative values indicated slower or worse performance (e.g. error rate, reaction time).

#### Partial least square (PLS)

To study co-variation between cognitive and brain changes across mid- and older adulthood, we used partial least squares, a multivariate method that sorts modes of common variation according to their brain-cognition covariance explained. The Canonical Partial Least Square (PLS) approach^33^, implemented in the Python library scikit-learn^61^, was used. Two datasets are given as inputs: the first contains sulcal anatomy measures (CT or SW of each sulcus), and the second comprises the individual cognitive tests. One latent variable is calculated for each dataset so that the covariance between them is maximized. The method iteratively calculates several pairs of latent variables. The first (principle) mode corresponds to the pair explaining the most covariance, and so on for the ensuing pairs. We refer to the pairs of latent variables as brain or cognitive projections. After assessing the robustness of each mode through permutation tests (see below), the contribution of each individual score (a specific cognitive test or sulci) to the shared variance is reflected in the corresponding loadings. Higher scores of these loadings correspond to better task performance and wider sulci, respectively.

As the default across the three data sets, we fitted the PLS models only to the healthy participants and permitted age effects to remain in the data. In auxiliary analyses on PISA participants, the effect of age and sex were first regressed out of each measure prior to fitting the PLS model on all participants (healthy, MCI and AD). To achieve this, a linear model with age and sex was fitted to predict each measure and the resulting prediction is then regressed, with PLS applied to the residuals.

For all analyses, missing values were replaced by the average of all participants (healthy or not) used to fit the PLS model. All measures were standardized by removing the mean of these same participants and scaling to unit variance before applying the PLS.

The code for this study is available at https://github.com/LeonieBorne/brain-cognition-pisa.

### Statistics

#### Permutation tests

PLS returns a series of modes, ranked by their covariance explained. Permutation tests were used to identify which of these modes were robust^62^. These tests consist of randomly shuffling subjects from one of the data domains (in this case, the cognitive measures dataset) to perturb the specific association with the other domain (MRI). Then PLS is re-performed and the covariance is measured between each pair of latent variables. This test is repeated 1000 times. If the covariance of any given mode obtained from the empirical data is greater than 95% of those obtained from the first mode with permutation tests then the mode is considered robust. As in Smith et al.^63^, we compared ensuing modes to the first mode because it extracts the highest explained variance in a null sample and can thus be viewed as the strictest measure of the null hypothesis^64^. To assess the significance of the original data against the permuted distribution, we use the covariance z-scored by the null distribution.

#### Bootstrapping

We used bootstrapping to identify which individual measures have a significant impact on the PLS latent variables^65^. This approach consists of creating a new database of the same size by randomly selecting participants with replacement. PLS is then performed on the bootstrapped data and the loadings between each initial measure and the corresponding latent variable are calculated. This test is repeated 1000 times. If the 2.5 and 97.5 percentiles of the loadings obtained have the same sign, the measure (a specific sulcus or cognitive measure) is considered to have a statistically significant impact on the calculation of the latent variable.

#### Statistical analyses

A series of statistical analyses were performed to assess the impact of specific risk factors for dementia on the latent variables. The impact of age was assessed using a two-sided hypothesis test, using the Wald Test with the t-distribution as the test statistic. To test whether any age-related effect differs between subgroups (diagnosis, sex, amyloid or *APOE* status), we used an analysis of covariance (ANCOVA) testing the interaction effect. The effect of sex (male, female) was evaluated using an ANCOVA, controlling for age. The effect of diagnoses (healthy, MCI, AD), amyloid status (positive, negative), *APOE* status (presence of the ε4 allele or not), and years of education (above or below the median for healthy participants) were evaluated using an ANCOVA controlling for age and sex. Because the PISA contains 16 twins, we removed one of each twin before using the ANCOVA to compare *APOE* status and controlled for age and sex. The PISA sample was enriched for high genetic risk of AD, including *APOE* ε4 positive as well as those in the highest quantile of risk for AD as defined by a polygenic risk score (PRS) combining common AD genetic risk variants with APOE ε4 omitted^32^. To control for any selection bias caused by APOE ε4 negative participants being enriched for other AD genetic risk variants, we also controlled the ANCOVA for the AD PRS used in the participant selection. Cross-cohort correlations of brain—loadings were calculated using Pearson correlations. In all three samples, a Bonferroni correction was applied by dividing the initial *p*-value of 0.05 by the number of tests (n = 14), resulting in a statistical threshold corrected for multiple comparisons of *p* < 0.004.

## Supplementary Information

Supplementary Information.

## Data Availability

Following completion of each wave (baseline, follow-up) and appropriate quality control, de-identified data will be made available to other research groups upon request. Due to privacy, confidentiality and constraints imposed by the local Human Research Ethics Committee, a “Data Sharing Agreement” will be required before data will be released. Due to ethics constraints, data will be shared on a project-specific basis. Depending on the nature of the data requested, evidence of local ethics approval may be required. For PISA data please contact michelle.lupton@qimrberghofer.edu.au, for AIBL and ADNI data please contact jurgen.mejan-fripp@csiro.au.
