# Supplementary material for: The interplay of age, gender and amyloid on brain and cognition in mid-life and older adults
Source: Sci Rep. 2024 Nov 8;14:27207. doi: 10.1038/s41598-024-78308-3 (PMC11549469; doi:10.1038/s41598-024-78308-3)
Supplement: Supplementary file 1 — Supplementary Information. [file 41598_2024_78308_MOESM1_ESM.docx]

# Supplementary material – Figures

**
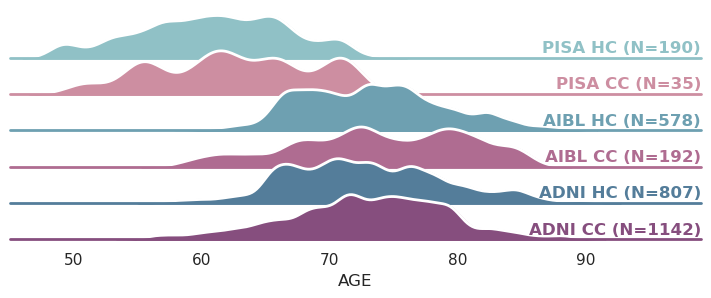
**

**Supplementary Figure 1: Age distribution for the three databases (PISA, AIBL and ADNI)**, each comprising a healthy cohort (HC) and a clinical cohort (CC) containing participants with MCI or AD. HC and CC participants in each database were matched for age and sex.

##


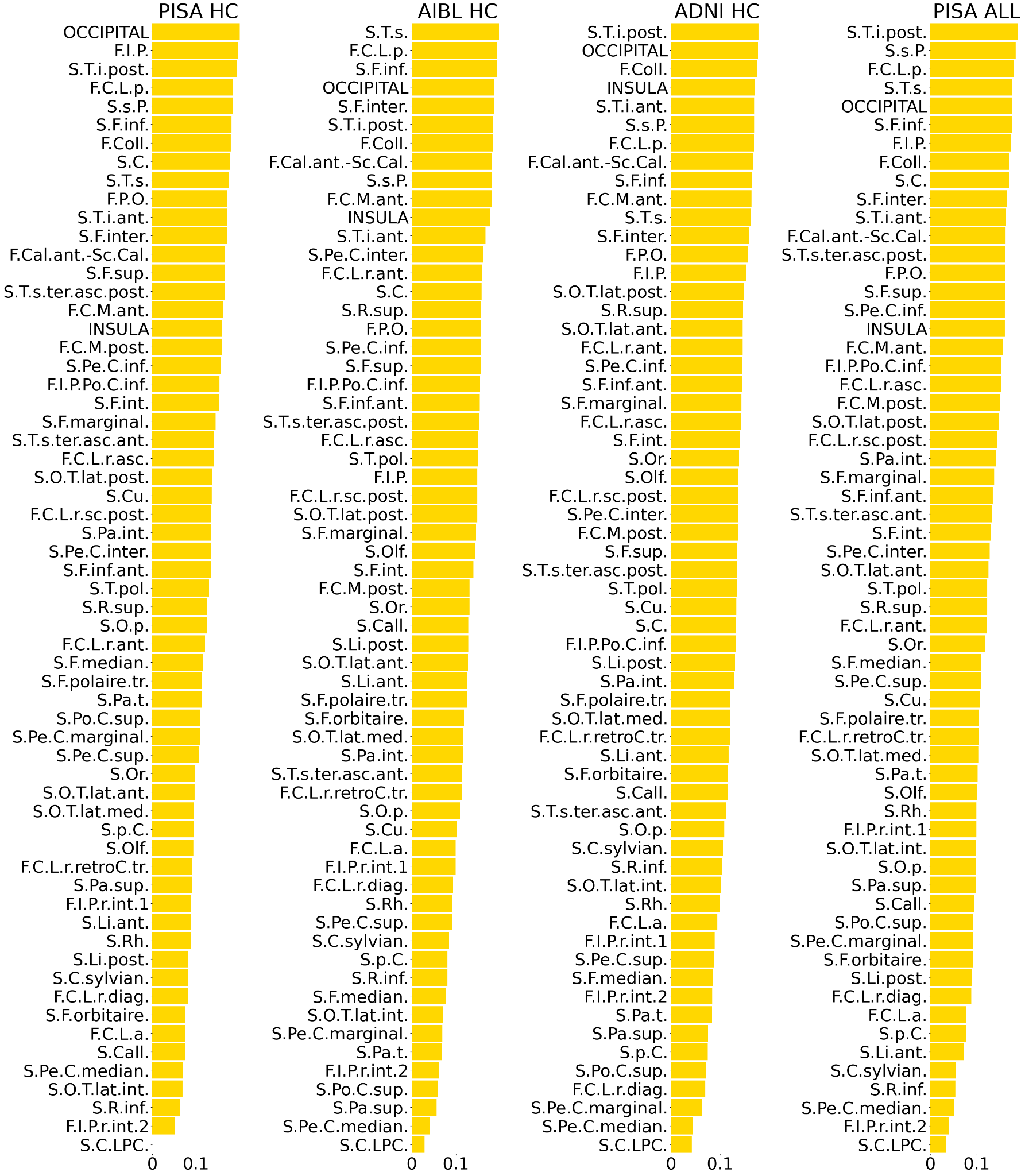


**Supplementary Figure 2:** Mean loading of all reliable cortical sulci for the robust PLS mode trained using sulcal width on PISA healthy cohort (PISA HC), AIBL healthy cohort (AIBL HC), ADNI healthy cohort (ADNI HC) and all PISA participants, after regression out of age and sex (PISA ALL).

**
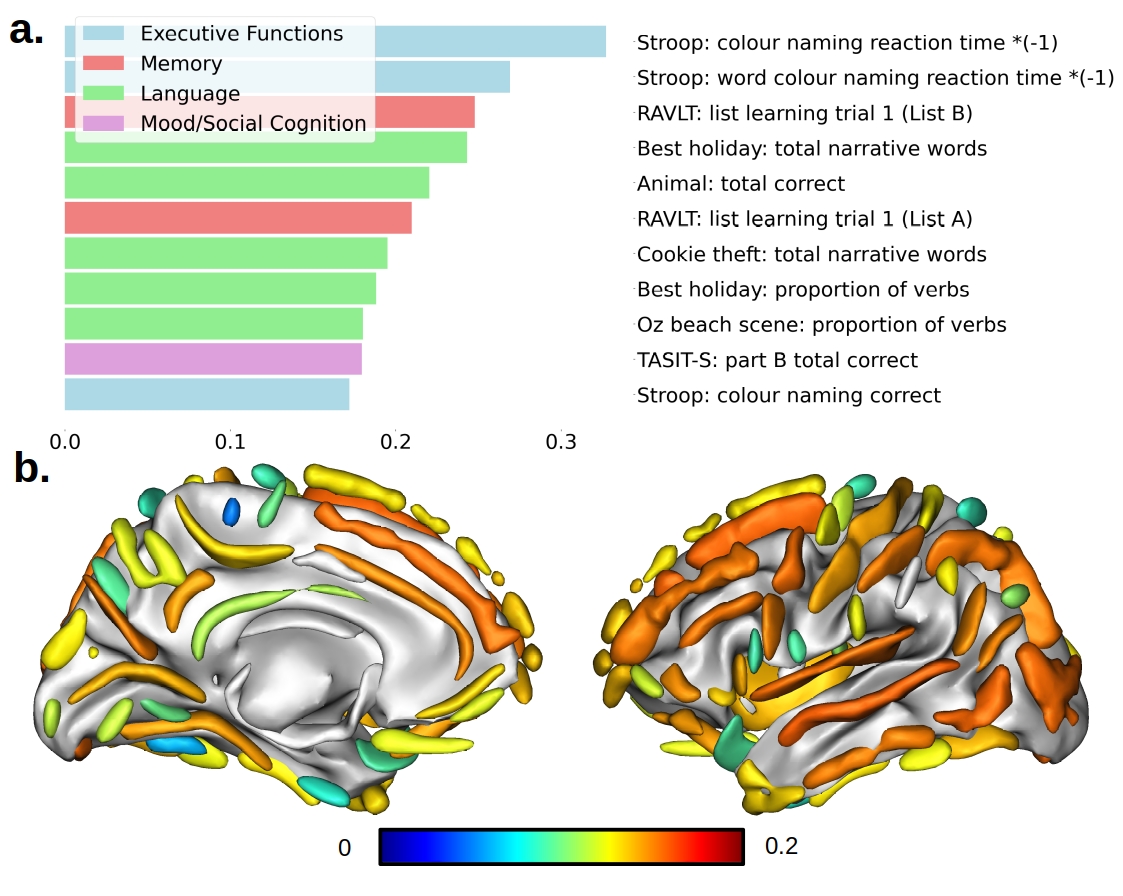
**

**Supplementary Figure 3: Robust PLS mode trained on PISA healthy cohort using cortical thickness.** (a) Mean loading of all reliable cognitive scores. (b) Mean loading of all reliable cortical sulci.

**
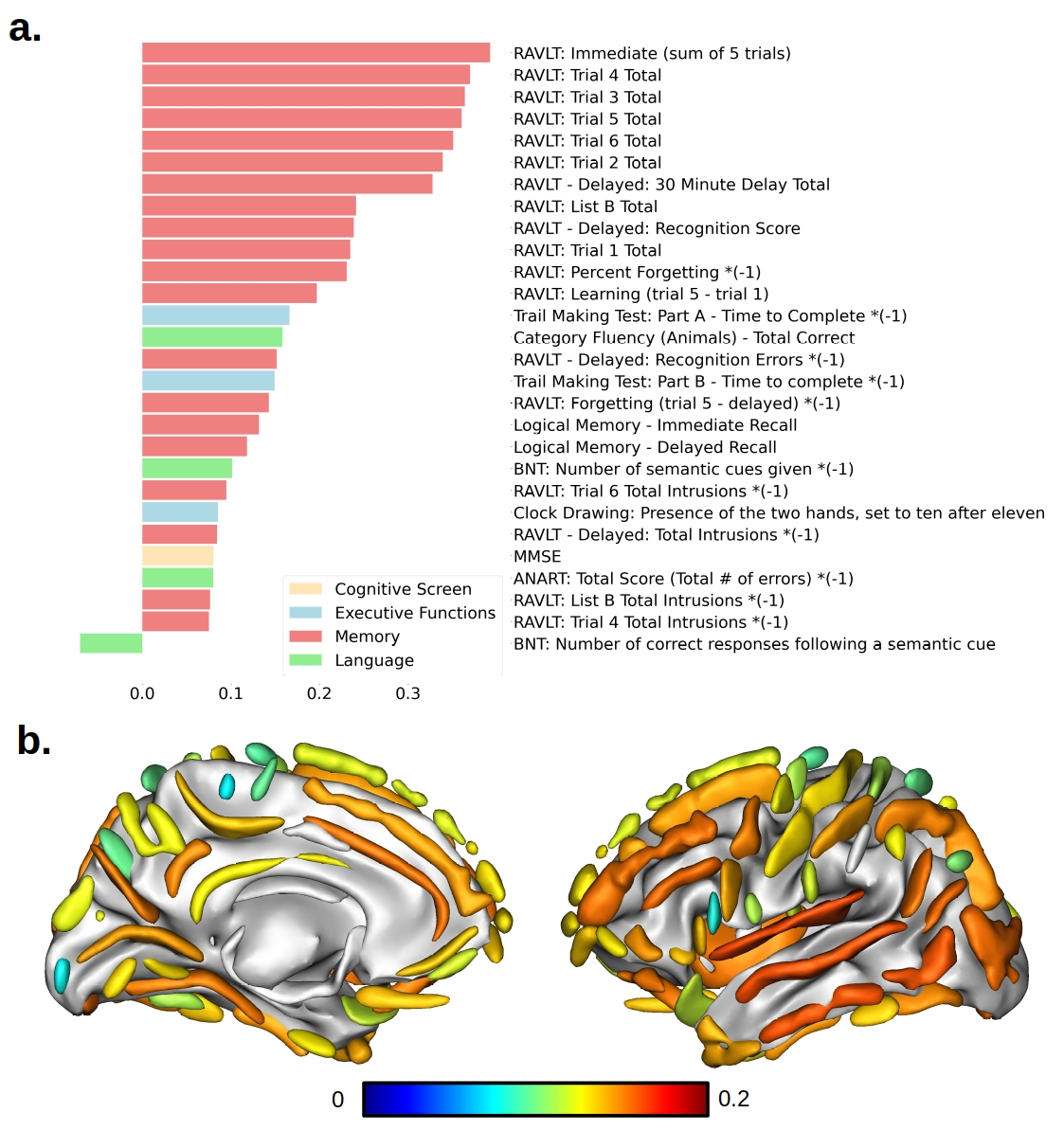
**

**Supplementary Figure 4: Robust PLS mode trained on ADNI healthy cohort using cortical thickness.** (a) Mean loading of all reliable cognitive scores. (b) Mean loading of all reliable cortical sulci.


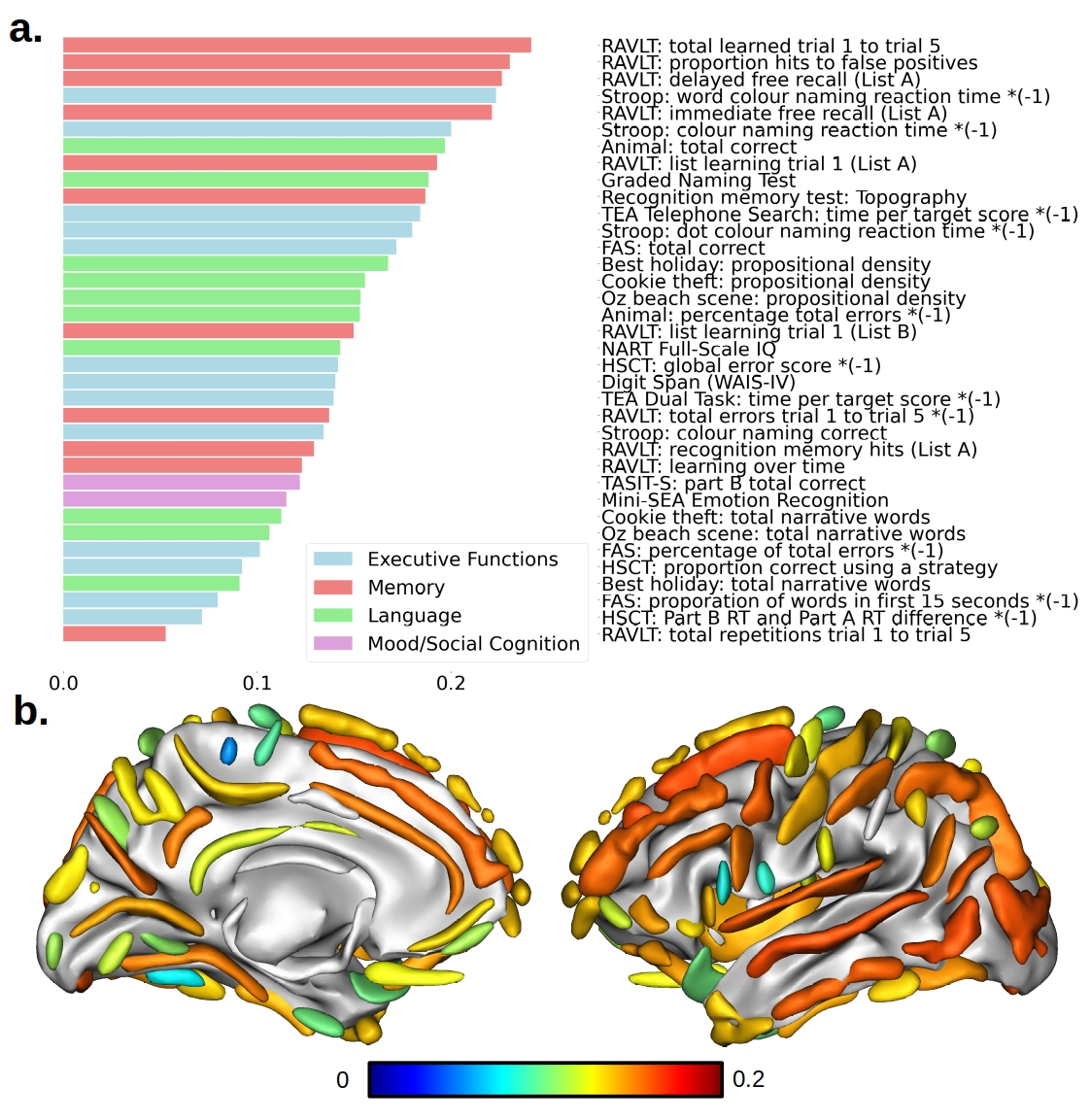


**Supplementary Figure 5: Loadings for PLS trained on PISA healthy and clinical cohort, using CT, after regressing out the age and sex of the participants.** (a) Mean loading of all reliable cognitive scores. (b) Mean loading of all reliable cortical sulci.


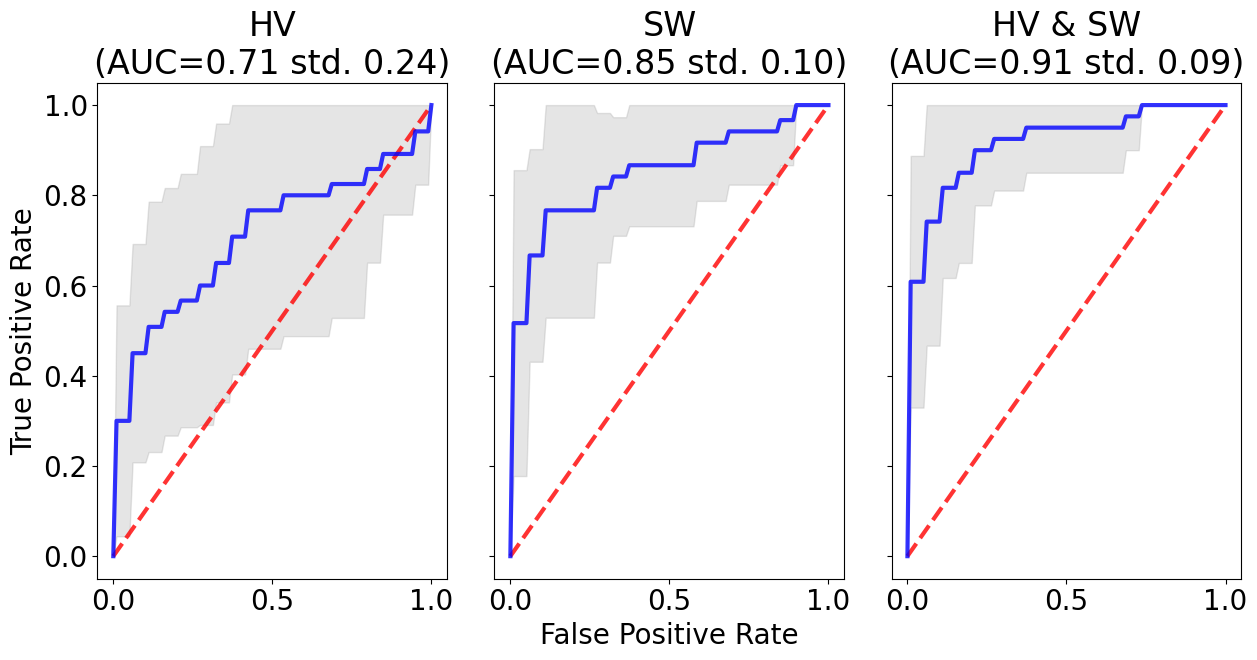


**Supplementary Figure 6:** **Healthy and clinical cohorts classification based on hippocampal volume (HV) or sulcal width (SW) projections from the PLS models trained on the healthy PISA cohort.** Compared to Figure 7d, the classification models also include age, sex, year of education and *APOE* status. Three categories are considered for *APOE* status (ɛ4 carrier, ɛ4 non-carrier, missing value) and are included as dummy variables.

**Supplementary Figure 7: Specific versus non-specific age and diagnostic effects.** Each column represents a different cohort. Results for the cognitive (a) and brain (b) projections for HC, MCI and AD participants, also benchmarked against randomly chosen features (grey). The violin plots represent the distribution of scores for 1000 permutation tests (bars represent the minimum, median and maximum value). The red line represents the original scores using PLS. The grey lines represent the scores for 100 representative permutations. The p-value is shown under the cohort label. Note that the p-value is not shown for the mean value of the healthy cohorts because the mean is always zero.

(a)

(b)


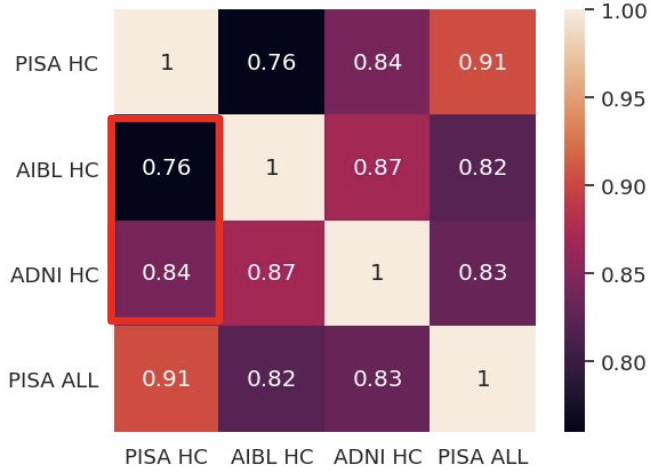


**Supplementary Figure 8: Age-independent Cross-Cohort correlation.** Heatmap of Pearson correlations for age-matched samples (median age 65) of healthy controls (HC) of the PISA, ADNI and AIBL cohorts as well as the full sample including healthy controls, MCI and AD of the PISA cohort.

**
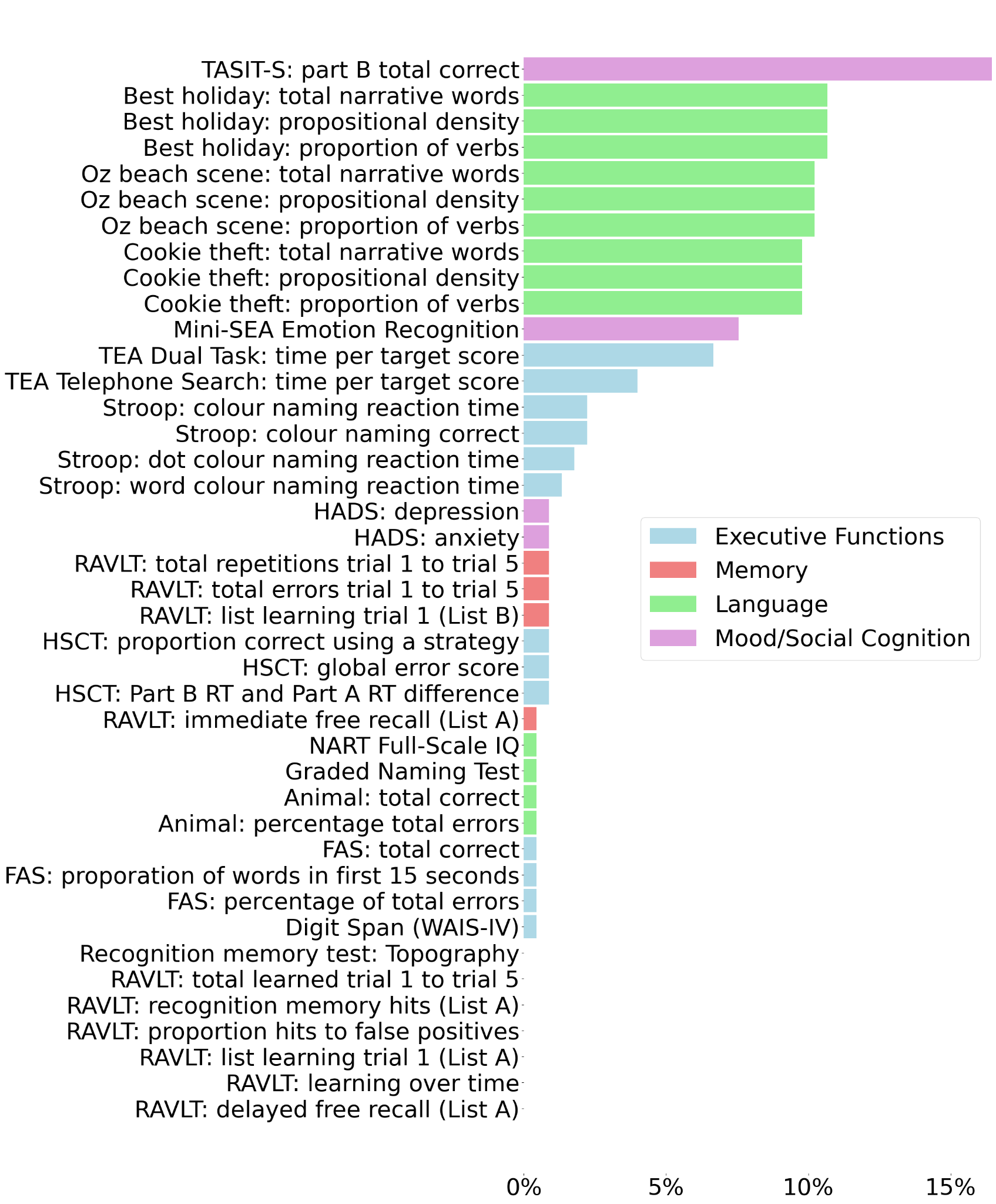
**

**Supplementary Figure 9: Percentage of missing values for PISA cognitive and mood assessments.**


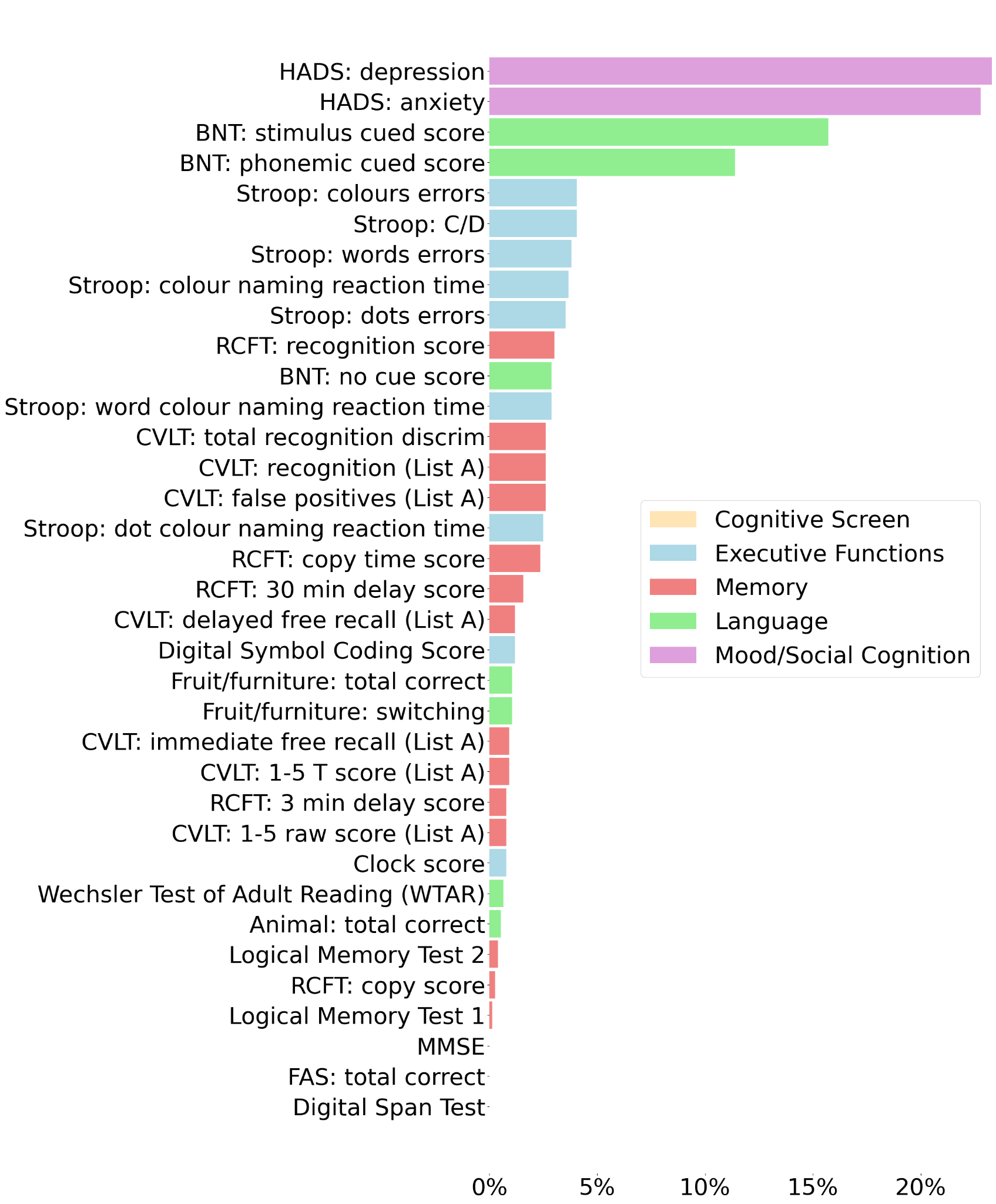


**Supplementary Figure 10: Percentage of missing values for AIBL cognitive and mood assessments.**

**
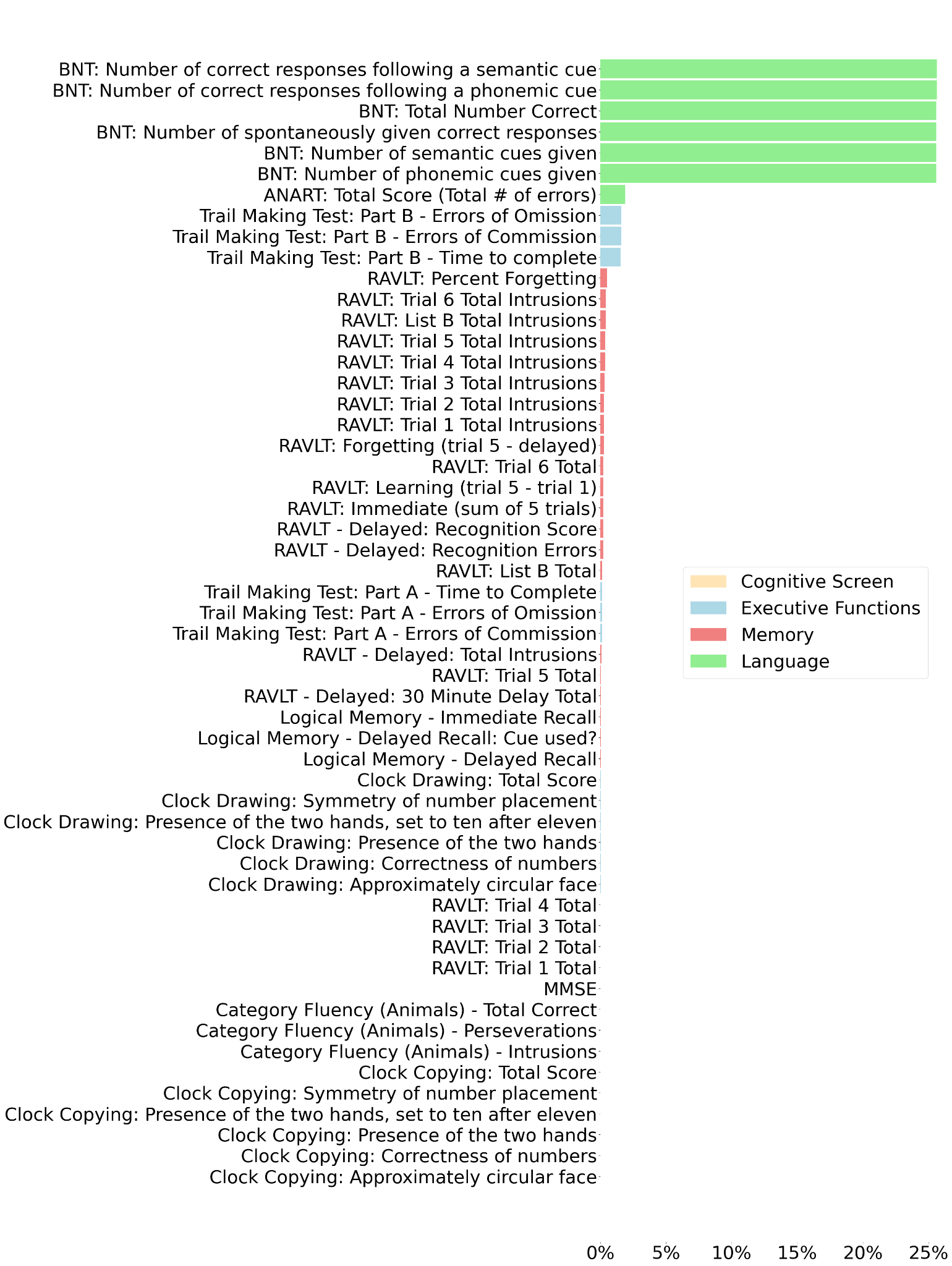
**

**Supplementary Figure 11: Percentage of missing values for ADNI cognitive and mood assessments.**

**
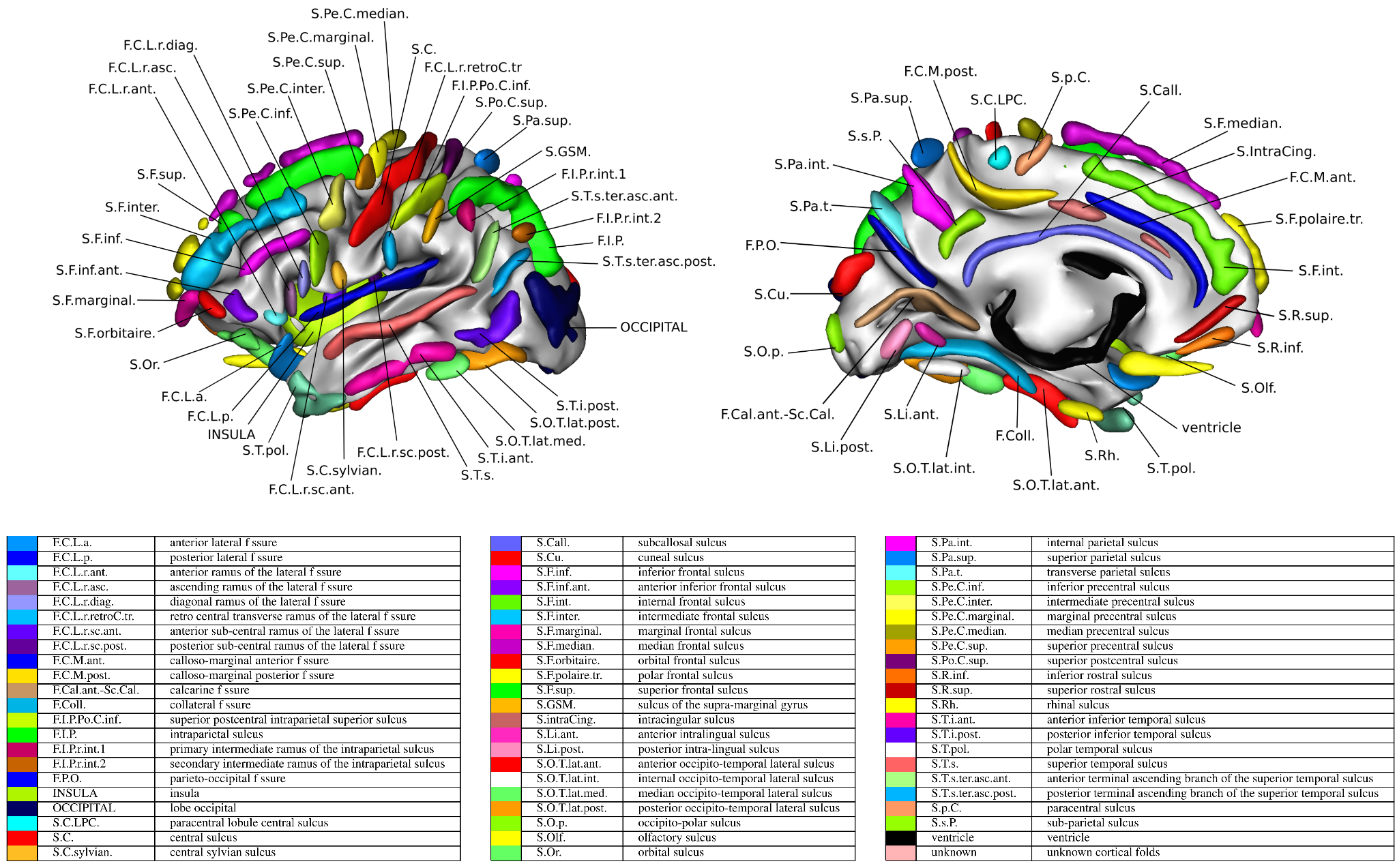
**

**Supplementary Figure 12. BrainVISA Sulci Atlas^1^**

# Supplementary material - Tables

**Supplementary Table 1. Database demographics.** An independent T-test was used to test the difference in mean age between the Healthy Cohort (HC) and the Clinical Cohort (CC). A Fisher exact test was used to test the difference in the proportion of male participants between the two cohorts.

|  | **PISA** | | **AIBL** | | **ADNI** | |
| --- | --- | --- | --- | --- | --- | --- |
|  | **HC** | **CC** | **HC** | **CC** | **HC** | **CC** |
| **N** | 190 | 35 | 573 | 191 | 807 | 1139 |
| **Mean age [range]** | 61 [49-73] | 63 [51-72] | 73 [60-89] | 74 [59-85] | 73 [57-90] | 73 [56-89] |
| T-test (age) | *p* = 0.10, *stat* = -1.7 | | *p* = 0.39, *stat* = -0.86 | | *p* = 0.99, *stat* = -0.0097 | |
| **Males N** | 49 | 15 | 255 | 101 | 356 | 554 |
| Fisher exact test (sex) | *p* = 0.065,  *odds-ratio* = 0.46 | | *p* = 0.054,  *odds-ratio* = 0.71 | | *p* = 0.053,  *odds-ratio* = 0.83 | |

**Supplementary Table 2.** Grouping of PISA, AIBL and ADNI neuropsychology assessments into four domains.

|  | **PISA** | **AIBL** | **ADNI** |
| --- | --- | --- | --- |
| **Cognitive Screen** |  | Mini-mental State Examination (MMSE) total score^2^ | Mini-mental State Examination (MMSE) total score^2^ |
| **Memory** | Rey Auditory Verbal Learning Test - Immediate and Delayed ^3,4^ | California Verbal Learning Test –Second edition (CVLT-II) ^5^ | Rey Auditory Verbal Learning Test - Immediate and Delayed ^4^ |
|  | Topographical Recognition Memory Test ^6^ |  |  |
|  |  | Logical Memory I and II (WMS; Story A only) ^7^ | Logical Memory II subscale of the Wechsler Memory Scale–Revised ^7^ |
|  |  | Rey Complex Figure Test (RCFT) ^8^ |  |
| **Executive Functions** | Stroop Test (Victoria version) ^9^ | Stroop task (Victoria version) ^9^ |  |
|  | Word fluency (FAS; ^10^) | D-KEFS verbal fluency - FAS ^11^ |  |
|  | Digit Span F/B (Wechsler Adult Intelligence Scale - Fourth edition - WAIS-IV;^12^) | Digit Span and Digit Symbol-Coding subtests of the Wechsler Adult Intelligence Scale – Third edition (WAIS–III) ^13^ |  |
|  | Hayling Sentence Completion Test ^14^;  Test of Everyday Attention: Telephone Search; Dual Task ^15^ |  |  |
|  |  | Clock Drawing Test ^16^ | Clock Drawing and Copying Tests ^16^ |
|  |  |  | Trail Making Test parts A and B ^17^ |
| **Language** | Graded Naming Test ^18^ | 30-item Boston Naming Test (BNT) ^19^ | Boston Naming Test ^20^ |
|  | National Adult Reading Test ^21^ | Wechsler Test of Adult Reading ^22^ | American National Adult Reading Test ^23^ |
|  | Spontaneous speech - complex scene description ^24^ |  |  |
|  | Category fluency - Animals ^10^ | D-KEFS verbal fluency ^11^ | Category Fluency - Animals ^25^ |
| **Mood & Social Cognition** | Hospital Anxiety and Depression Scale (HADS) ^26^ | Hospital Anxiety and Depression Scale (HADS) ^26^ |  |
|  | TASIT-S part B Sarcasm ^27^ |  |  |
|  | Mini-SEA emotion evaluation ^28^ |  |  |

# Supplementary material - Method

**Supplementary method - Brain imaging acquisition**

*PISA:* Structural data were acquired using a 3D T1 MPRAGE sequence (TE/TR=2.26 ms/2.3 s, TI=0.9 s, FA=8˚, 1 mm isotropic resolution, matrix 256 x 240 x 192, BW=200 Hz/Px, 2x GRAPPA acceleration) at 3T on a Biograph mMR hybrid scanner (Siemens Healthineers, Erlangen, Germany). PET data were acquired on the same scanner with Fluorine-18 florbetaben ([^18^F]FBB), a diagnostic radiotracer which possesses a highly selective binding for β-amyloid in neural tissue ^29, 30^.

*AIBL & ADNI:* For AIBL, structural data were acquired on 7 different Siemens scanners using a 3D T1 MPRAGE sequence at 3T (79%) or at 1.5T (21%). For ADNI. structural data were acquired on 65 different sites using a 3D T1 MPRAGE sequence at 3T (66%) or at 1.5T (34%). For both databases, amyloid data were assessed with Pittsburgh Compound B, an in vivo amyloid imaging tracer ^31^.

**Supplementary method - Brain imaging preprocessing**

*Quality Control measures:* For PISA quality control pipelines have been described in the appendices of Lupton et al., 2021^32^ and includes the use of the MP2RAGE Bloch equations^33^, and cortical thickness computed using a Lagrangian-Eulerian PDE approach^34^, ADNI data underwent a 2-step quality control that is described at https://adni.loni.usc.edu/data-samples/adni-data/neuroimaging/mri/mri-quality-control/ and involves an automatized check for adherence to protocol parameters and a trained analyst that manually inspects images to ensure series-specific quality, and assigns a numerical grade from 1-3, including factors such as motion artifacts, anatomical coverage, completeness of the scan, and overall image quality. AIBL quality control is described in Ellis and colleagues^35^.

*Sulcal measurements:* The preprocessing of T1 structural MRIs is performed using the Morphologist pipeline of the BrainVISA toolbox. BrainVISA applies a spatial bias correction^36^, utilizing a fully automated method to correct intensity nonuniformity in MR images, thereby restoring a meaningful relationship between intensity and tissue classification. This correction addresses spatial inhomogeneities caused by limitations in the acquisition process. The method involves estimating a smooth multiplicative field that minimizes the entropy of the intensity distribution^37^, followed by an analysis to infer the statistical properties of grey and white matter. Based on this analysis, the image is binarized to isolate the intensity range corresponding to the brain. A simple Markovian regularization enhances the robustness of this step. Mathematical morphology is then employed to create a brain mask by eroding the binary image until the largest connected component no longer intersects a 5 mm layer from the outer surface of the head. Finally, the brain shape is reconstructed through conditional dilation of this seed ^38^.A scale-space based approach provides robustness to the various variations observed across MR sequences and subjects , analyzing the T1-weighted histogram to estimate grey/white statistics, computing a binary mask of the brain from the bias corrected T1-weighted image^38^, splitting the brain mask in 3 parts (hemispheres and cerebellum), creating the grey/white interface and automatically labelling the cortical folds using the deep learning model^1^.

*Hippocampal Volume:* The hippocampal volumes were calculated using the CurAIBL (Computational qUantification of mRi from AIBL) computing platform^39^. In this pipeline, MRIs are first rigidly registered to the MNI average brain, and segmented into grey and white matter and CSF using Expectation Maximisation Segmentation algorithm. The images are then parcellated using the 20 most similar atlases, selected from a database of 843 images. Hippocampus volume is extracted using the Harmonized Protocol for Hippocampal Volumetry.

##

## Supplementary material - References

1. Borne, L., Rivière, D., Mancip, M., Mangin, J.-F., 2020. Automatic labeling of cortical sulci using patch- or CNN-based segmentation techniques combined with bottom-up geometric constraints. Med. Image Anal. 62, 101651. <https://doi.org/10.1016/j.media.2020.101651>
2. Folstein, M.F., Folstein, S.E., McHugh, P.R., 1975. “Mini-mental state.” J. Psychiatr. Res. 12, 189–198. <https://doi.org/10.1016/0022-3956(75)90026-6>
3. Ivnik, R.J., Malec, J.F., Tangalos, E.G., Petersen, R.C., Kokmen, E., Kurland, L.T., 1990. The Auditory-Verbal Learning Test (AVLT): Norms for ages 55 years and older. Psychol. Assess. J. Consult. Clin. Psychol. 2, 304–312. <https://doi.org/10.1037/1040-3590.2.3.304>
4. Rey, A., 1964. Auditory verbal learning test. Psychol. Apprais. Child. Cereb. Deficits Camb. Mass. Harv. Univ. Press I96 I.
5. Delis, D.C., Kramer, J.H., Kaplan, E., Ober, B.A., 2000. California verbal learning test-second edition. Adult Version Man. Psychol. Corp.
6. Warrington, E.K., 1996. The Camden Memory Tests Manual. Psychology Press.
7. Wechsler, D., 1945. A Standardized Memory Scale for Clinical Use. J. Psychol. 19, 87–95. https://doi.org/10.1080/00223980.1945.9917223
8. Meyers, J.E., Meyers, K.R., 1995. Rey complex figure test under four different administration procedures. Clin. Neuropsychol. 9, 63–67. https://doi.org/10.1080/13854049508402059
9. Troyer, A.K., Leach, L., Strauss, E., 2006. Aging and Response Inhibition: Normative Data for the Victoria Stroop Test. Aging Neuropsychol. Cogn. 13, 20–35. https://doi.org/10.1080/138255890968187
10. Tombaugh, T.N., Kozak, J., Rees, L., 1999. Normative Data Stratified by Age and Education for Two Measures of Verbal Fluency: FAS and Animal Naming. Arch. Clin. Neuropsychol. 14, 167–177. https://doi.org/10.1093/arclin/14.2.167
11. Delis, D.C., Kaplan, E., Kramer, J.H., 2001. Delis-Kaplan executive function system.
12. Wechsler, D., 2008. Wechsler adult intelligence scale–Fourth Edition (WAIS–IV). San Antonio TX NCS Pearson 22, 816–827.
13. Wechsler, D., 1955. Wechsler adult intelligence scale--. Arch. Clin. Neuropsychol.
14. Burgess, P.W., Shallice, T., 1997. The hayling and brixton tests.
15. Robertson, I.H., Ward, T., Ridgeway, V., Nimmo-Smith, I., 1994. The test of everyday attention (TEA). Bury St Edmunds UK Thames Val. Test Co. 197–221.
16. Goodglass, H., Kaplan, E., 1972. The assessment of aphasia and related disorders. Lea & Febiger.
17. Reitan, R.M., 1958. Validity of the Trail Making Test as an indicator of organic brain damage. Percept. Mot. Skills 8, 271–276.
18. Warrington, E.K., 1997. The Graded Naming Test: A Restandardisation. Neuropsychol. Rehabil. 7, 143–146. https://doi.org/10.1080/713755528
19. Saxton, J., Ratcliff, G., Munro, C.A., Coffey, E.C., Becker, J.T., Fried, L., Kuller, L., 2000. Normative Data on the Boston Naming Test and Two Equivalent 30-Item Short Forms. Clin. Neuropsychol. 14, 526–534. https://doi.org/10.1076/clin.14.4.526.7204
20. Goodglass, H., Kaplan, E., Weintraub, S., 1983. Boston naming test. Lea & Febiger Philadelphia, PA.
21. Nelson, H.E., Willison, J., 1991. National adult reading test (NART). Nfer-Nelson Windsor.
22. Wechsler, D., 2001. Wechsler Test of Adult Reading: WTAR. Psychological Corporation.
23. Taylor, K.I., Salmon, D.P., Rice, V.A., Bondi, M.W., Hill, L.R., Ernesto, C.R., Butters, N., 1996. Longitudinal examination of american national adult reading test (AMNART) performance in dementia of the Alzheimer type (DAT): Validation and correction based on degree of cognitive decline. J. Clin. Exp. Neuropsychol. 18, 883–891. https://doi.org/10.1080/01688639608408309
24. Robinson, G.A., Spooner, D., Harrison, W.J., 2015. Frontal dynamic aphasia in progressive supranuclear palsy: Distinguishing between generation and fluent sequencing of novel thoughts. Neuropsychologia 77, 62–75. https://doi.org/10.1016/j.neuropsychologia.2015.08.001
25. Morris, J.C., Heyman, A., Mohs, R.C., Hughes, J.P., van Belle, G., Fillenbaum, G., Mellits, E.D., Clark, C., 1989. The consortium to establish a registry for Alzheimer’s disease (CERAD): I. Clinical and neuropsychological assessment of Alzheimer’s disease. Neurology 39, 1159–1165. https://doi.org/10.1212/WNL.39.9.1159
26. Snaith, R.P., Zigmond, A.S., 1986. The hospital anxiety and depression scale. Br. Med. J. Clin. Res. Ed 292, 344.
27. Mcdonald, S., Flanagan, S., Honan, C., 2017. The Awareness of Social Inference TEST - Short (TASIT-S) Manual.
28. Bertoux, M., Volle, E., de Souza, L.C., Funkiewiez, A., Dubois, B., Habert, M.O., 2014. Neural correlates of the mini-SEA (Social cognition and Emotional Assessment) in behavioral variant frontotemporal dementia. Brain Imaging Behav. 8, 1–6. https://doi.org/10.1007/s11682-013-9261-0
29. Fodero-Tavoletti, M.T., Brockschnieder, D., Villemagne, V.L., Martin, L., Connor, A.R., Thiele, A., Berndt, M., McLean, C.A., Krause, S., Rowe, C.C., Masters, C.L., Dinkelborg, L., Dyrks, T., Cappai, R., 2012. In vitro characterization of [18F]-florbetaben, an Aβ imaging radiotracer. Nucl. Med. Biol. 39, 1042–1048. https://doi.org/10.1016/j.nucmedbio.2012.03.001
30. Rowe, C.C., Ackerman, U., Browne, W., Mulligan, R., Pike, K.L., O’Keefe, G., Tochon-Danguy, H., Chan, G., Berlangieri, S.U., Jones, G., Dickinson-Rowe, K.L., Kung, H.P., Zhang, W., Kung, M.P., Skovronsky, D., Dyrks, T., Holl, G., Krause, S., Friebe, M., Lehman, L., Lindemann, S., Dinkelborg, L.M., Masters, C.L., Villemagne, V.L., 2008. Imaging of amyloid β in Alzheimer’s disease with 18F-BAY94-9172, a novel PET tracer: proof of mechanism. Lancet Neurol. 7, 129–135. https://doi.org/10.1016/S1474-4422(08)70001-2
31. Pike, K.E., Savage, G., Villemagne, V.L., Ng, S., Moss, S.A., Maruff, P., Mathis, C.A., Klunk, W.E., Masters, C.L., Rowe, C.C., 2007. β-amyloid imaging and memory in non-demented individuals: evidence for preclinical Alzheimer’s disease. Brain 130, 2837–2844. https://doi.org/10.1093/brain/awm238
32. Lupton, M. K. et al. A prospective cohort study of prodromal Alzheimer’s disease: Prospective Imaging Study of Aging: Genes, Brain and Behaviour (PISA). NeuroImage Clin. 29, 102527 (2021).
33. Duche, Q., Saint-Jalmes, H., Acosta. O., Raniga, P., Bourgeat, P., Dore, V., Egan, G.F., Salvado, O. 2017. Partial volume model for brain MRI scan using MP2RAGE. Human Brain Mapping 38(10), 5115-5127.
34. Acosta, O., Bourgeat, P., Zuluaga, M.A., Jurgen, F., Salvado, O., Ourselin, S., Alzheimers Disease Neuroimaging, I. 2009. Automated voxel-based 3D cortical thickness measurement in a combined Lagrangian-Eulerian PDE approach using partial volume maps. Med Image Anal 13(5), 730-743.
35. Ellis, K. A., Bush, A. I., Darby, D., De Fazio, D., Foster, J., Hudson, P., ... & AIBL Research Group. (2009). The Australian Imaging, Biomarkers and Lifestyle (AIBL) study of aging: methodology and baseline characteristics of 1112 individuals recruited for a longitudinal study of Alzheimer's disease. *International psychogeriatrics*, *21*(4), 672-687.
36. Mangin, J.F.,  *Riviere, D., Cachia, A., Duchesnay, E., Cointepas, Y., Papadopoulos-Orfanos, D., Collins, D.L., Evans, A.C., Regis, J. (2004).* Object-based morphometry of the cerebral cortex," in *IEEE Transactions on Medical Imaging*, vol. 23, no. 8, pp. 968-982, Aug. 2004, doi: 10.1109/TMI.2004.831204.
37. Mangin, J.-F., 2000. Entropy minimization for automatic correction of intensity nonuniformity, in: Proceedings IEEE Workshop on Mathematical Methods in Biomedical Image Analysis. MMBIA-2000 (Cat. No.PR00737). Presented at the Proceedings IEEE Workshop on Mathematical Methods in Biomedical Image Analysis. MMBIA-2000 (Cat. No.PR00737), pp. 162–169. <https://doi.org/10.1109/MMBIA.2000.852374>
38. Mangin, J.-F., Coulon, O., Frouin, V., 1998. Robust brain segmentation using histogram scale-space analysis and mathematical morphology, in: Wells, W.M., Colchester, A., Delp, S. (Eds.), Medical Image Computing and Computer-Assisted Intervention — MICCAI’98, Lecture Notes in Computer Science. Springer, Berlin, Heidelberg, pp. 1230–1241. https://doi.org/10.1007/BFb0056313
39. Bourgeat, P., Doré, V., Fripp, J., Ames, D., Masters, C.L., Rowe, C.C., Salvado, O., 2015. IC-P-130: Web-based automated PET and MR quantification. Alzheimers Dement. 11, P88–P88. https://doi.org/10.1016/j.jalz.2015.06.152
